# Phytochemistry and Diverse Pharmacology of Genus *Mimosa*: A Review

**DOI:** 10.3390/biom12010083

**Published:** 2022-01-05

**Authors:** Komal Rizwan, Ismat Majeed, Muhammad Bilal, Tahir Rasheed, Ahmad Shakeel, Shahid Iqbal

**Affiliations:** 1 Department of Chemistry, University of Sahiwal, Sahiwal 57000, Pakistan; 2 Department of Chemistry, Government College Women University, Faisalabad 38000, Pakistan; ismatmajeed123@gmail.com; 3 School of Life Science and Food Engineering, Huaiyin Institute of Technology, Huaian 223003, China; bilaluaf@hyit.edu.cn; 4 Interdisciplinary Research Center for Advanced Materials, King Fahd University of Petroleum and Minerals (KFUPM), Dhahran 31261, Saudi Arabia; masil@sjtu.edu.cn; 5 Department of Hydraulic Engineering, Faculty of Civil Engineering and Geosciences, Delft University of Technology, Stevinweg 1, 2628 CN Delft, The Netherlands; 6 Department of Chemistry, School of Natural Sciences (SNS), National University of Science and Technology (NUST), H-12, Islamabad 46000, Pakistan; shahidiqbal.chem@sns.nust.edu.pk

**Keywords:** genus, *Mimosa*, plant, phytochemicals, biological molecules, pharmacological activities

## Abstract

The genus *Mimosa* belongs to the Fabaceae family and comprises almost 400 species of herbs, shrubs and ornamental trees. The genus *Mimosa* is found all over the tropics and subtropics of Asia, Africa, South America, North America and Australia. Traditionally, this genus has been popular for the treatment of jaundice, diarrhea, fever, toothache, wound healing, asthma, leprosy, vaginal and urinary complaints, skin diseases, piles, gastrointestinal disorders, small pox, hepatitis, tumor, HIV, ulcers and ringworm. The review covered literature available from 1959 to 2020 collected from books, scientific journals and electronic searches, such as Science Direct, Web of Science and Google scholar. Various keywords, such as *Mimosa*, secondary metabolites, medicines, phytochemicals and pharmacological values, were used for the data search. The *Mimosa* species are acknowledged to be an essential source of secondary metabolites with a wide-ranging biological functions, and up until now, 145 compounds have been isolated from this genus. Pharmacological studies showed that isolated compounds possess significant potential, such as antiprotozoal, antimicrobial, antiviral, antioxidant, and antiproliferative as well as cytotoxic activities. Alkaloids, chalcones, flavonoids, indoles, terpenes, terpenoids, saponins, steroids, amino acids, glycosides, flavanols, phenols, lignoids, polysaccharides, lignins, salts and fatty esters have been isolated from this genus. This review focused on the medicinal aspects of the *Mimosa* species and may provide a comprehensive understanding of the prospective of this genus as a foundation of medicine, supplement and nourishment. The plants of this genus could be a potential source of medicines in the near future.

## 1. Introduction

The *Mimosa* genus is a member of the legumes family Fabaceae (subfamily: *mimosoideae*) and consists of about 400 species of shrubs and herbs [1]. Almost 20–25 species of this genus are well known to the world including *Mimosa tenuiflora* (Wild.) pior, *Mimosa pudica* L., *Mimosa pigra* L., *Mimosa caesalipiniifolia* Benth., *Mimosa hamata* Willd., *Mimosa diplotricha* Sauvalle, *Mimosaa xanthocentra* Mart., *Mimosa artemisiana* Heringer & Paula, *Mimosa invisa* Mart. ex Colla, *Mimosa scabrella* Benth., *Mimosa somnians* Humb. & Bonpl. ex Willd., *Mimosa bimucronata* (DC.) Kuntze, *Mimosa verrucosa* Benth., *Mimosa arenosa* (Willd.) Poir., *Mimosa humilis* Willd., *Mimosa rubicaulis* Lam., *Mimosa linguis* and *M. albida* Willd., and *Mimosa ophthalmocentra* Mart. ex Benth. (http://mpns.kew.org/MPNS.kew.org accessed on 12 December 2018; www.theplantlist.org accessed on 12 December 2018). These species are found in Brazil [2], Africa (Mauritius, Nigeria and Reunion), numerous Pacific Islands, North America, Papua New Guinea [3], Australia [4], Mexico, Venezuela [5], Thailand and the Philippines [6]. However, they are mainly found in Asia, such as Pakistan [7,8], Japan, Indonesia, India, Bangladesh, Malaysia [9], India [10], China, Sri Lanka, Taiwan and Cambodia, while certain species are commonly distributed from Cuba to Texas, northern Central America, Paraguay, Uruguay and Argentina, [11]. The species of *Mimosa* grow in diverse habitats, such as the savannas, lowland tropical rainforests, dry forests and thorn scrubs of tropical and subtropical areas, midelevations of subtropical deserts, forests, wetlands and grasslands [12]. The leaves of the *Mimosa* species may be binate or pinnate. Some species such as *M. pudica* and *M. pigra* show response to touch by folding their leaves. Flowers may be white, globular pink and in the form of clusters. The fruits are brittle, and a wall of the fruit is compacted in the middle of the seeds. The root bark contains large amounts of starch and calcium oxalate crystals [13,14].

The plants of this genus especially are employed for ornamental purposes and also serve as a sleeping shelter for animals [15]. Various species of the *Mimosa* genus are socially and economically important, such as *M. scabrella* (timber production), *M. caesalpiniifolia* (reforestation) and *M. tenuiflora* (source of firewood). These plants are also used for flooring and furniture [16,17]. The leaves of the plants are used for chicken diet [18], as colorants in the textile industry and also play a role as additives in the food industry [19,20]. The trash of the poultry industry enhances the growth of the *Mimosa* tree [21,22]. The *Mimosa* genus has significant economic status in the cosmetic industry [23]. Phytochemical studies of this genus revealed the presence of flavonoids, steroids, saponins, alkaloids, coumarins, tannins [24,25] and terpenoids [26]. The genus *Mimosa* showed several pharmacological activities, such as antiseptic, antimicrobial [27,28], antidiabetic [29,30], antihistamic [31], antioxidants [32,33,34,35,36,37], anticonvulsant [38], antigout [39], antispasmolytic [40], anti-inflammatory [41,42,43], antinociceptive [44,45], antiulcer [43], antifertility [46], antimalarial [47], antiparasitic [48], wound healing [49], anticancer [50,51], antidepressant [52], antidiarrheal [53,54], hypolipidemic [55,56], hepatoprotective [57], antivenom [43], antiproliferative [58], antiviral [59] and aphrodisiac [60]. Previously our group efficiently documented the indigenous flora of Pakistan and also a few exotic species [61,62,63,64,65,66,67,68,69,70,71,72,73,74,75]. This review covered the several review articles as well in the discussion regarding this genus [76,77,78,79,80]. Recently, our group reported the ethotraditional uses and pharmacological potential of the crude extracts of different species of the genus *Mimosa* [81]. In this review, we comprehensively reported the secondary metabolites isolated from the genus *Mimosa* and their pharmacological potential along with future perspectives.

## 2. Materials and Methods

A detailed bibliographic study that included papers published from 1959 to 2020 was conducted. A number of databanks (Web of Science, Science Direct, SciFinder, Francis & Taylor, Scopus, SciELO, Google Scholar, Springer, PubMed, Wiley, Google and The Plant-Database) were surveyed for assembling statistics, data and figures for this genus. A number of related books, complete text documents and summaries were checked. The genus name, synonyms and scientific names of the genus *Mimosa* species were castoff as the keywords. The scientific name of all the plants of genus *Mimosa* and its substitutes were corroborated by consuming a standard databank (http://mpns.kew.org/MPNS.kew.org accessed on 12 December 2018; www.theplantlist.org accessed on 12 December 2018).

## 3. Chemical Profiling of Genus *Mimosa*

### 3.1. Qualitative and Quantitative Analysis of Phytochemicals in Genus Mimosa

Haddad et al. determined condensed tannins of *M. tenuiflora* whose active ingredients were procyanidin and prodelphinidins [82]. Oliveira et al. determined the total phenols (TP; 99.29 and 65.37), total tannins (TT; 65.57 and 54.93) and condensed tannins (CT; 34.56 and 30.98) from the leaves and stems of *M. tenuiflora,* respectively [83]. Racadio reported the existence of phytochemicals in the EtOH extract of *M. pudica* leaves. Phytochemicals, such as alkaloids, flavonoids, saponins and triterpenes, were present, while sterols and tannins were found to be absent [84]. Ahuchaogu et al. screened the phytochemicals of the EtOH extract of the whole *M. pudica* plant. The ethanol extract consisted of alkaloids (9.05%), flavonoids (8.32%), steroids (2.49%), saponins (8.15%), phenols (1.02%), tannins (0.083%), cyanogenic glycosides (0.122%) and anthocyanins (1.913%) [85]. Durgadevi and Karthika reported the phytochemicals of the aq. extract of *M. pudica* leaves. A qualitative analysis indicated the presence of flavonoids, alkaloids, proteins, steroids, tannins, saponins and terpenoids, while phlobatannins, phenols and reducing sugars were found to be absent. The quantitative phytochemical investigation of saponins, flavonoids, tannins and terpenoids were 0.48 mg/mL, 0.99 mg/mL, 0.80 mg/mL, 0.39 mg/mL and 0.39 mg/mL of the aq. extract recorded, respectively [24]. Sheeba et al. screened the phytochemicals of the MeOH extracts of *M. pudica* leaves. The plant showed the presence of flavonoids, phenols and tannins, while alkaloids, glycosides, terpenoids, amino acids and carbohydrates were found to be absent [86]. Tunna et al. reported the presence of phytochemicals in *M. pudica* aerial parts. Alkaloids, saponins, flavonoids, terpenoids and coumarins were found to be present, while carotenoids and anthraquinone were found to be absent [29]. Parmar et al. measured the phytochemicals of the EtOH extract of *M. pudica* roots. Extracts showed the presence of tannins, alkaloids, terpenoids, flavonoids, sterols, phenolic compounds and proteins [34]. *Mahadevan* et al. measured the phytochemicals of aq. extract of the whole *M. pudica* plant. A Phytochemical analysis exhibited the existence of alkaloids, flavonoids and tannins [87]. Ramesh et al. investigated phytochemicals of various extracts (EtOH, MeOH, PE and ACE) of *M. pudica* leaves and roots. The results showed the presences of flavonoids, alkaloids, terpenoids, carbohydrates, saponins, amino acids, phenols, tannins, proteins and steroids, while glycosides, fats, oils, resins, reducing sugars, phytosterols and phlobatannins were found to be absent [88]. Chinnathambi and Sathasivam measured the phytochemicals of the ACE, EtOH and aq. extracts of *M. pudica* leaves. The investigation proved the existence of tannins, terpenoids, phlobatannins, steroids, saponins and glycoside, while flavonoids were absent [89]. Mathew et al. measured the phytochemicals of the MeOH extracts of the *M. pudica* plant. The results showed the existence of compounds, such as flavonoids alkaloids, saponins, terpenoids, phenols, glycosides, tannins and coumarins [26]. Nagarajan et al. determined the phytochemicals of aq. extract of *M. pudica* leaves and stems by using the Harborne methods [90,91]. The phytochemicals, such as saponins, alkaloids, flavonoids, tannins and phenols were present.

Nagarajan et al. determined the quantitative assessment of the mineral contents of the aq. extracts of *M. pudica* leaves and stems by using wet digestion extraction methods. The mineral contents, such as magnesium, phosphorus, calcium, nitrogen and potassium, were observed [91]. Lee et al. reported the presence of neoxanthin (9.86 μgg^−1^ FW), viola xanthin (6.57 μgg^−1^ FW), lutein (7.75 μgg^−1^ FW), lycopene (0.62 μgg^−1^ FW), carotene (*α =* 0.19, *β* = 0.25 vit.E^−1^ g), tocopherol (*α =* 0.25 vit.E^−1^ g), total Carotenoids (25.24 μgg^−1^ FW) and total vitamins (0.25 μgg^−1^ FW) in leaves of *M. pudica* [92]. Ittiyavirah and Pullochal determined the phytochemicals of the EtOH extract of the whole *M. pudica* plant. The preliminary phytochemical analysis of *M. pudica* revealed the existence of alkaloids, flavonoids, tannins, phenolics and steroids [93]. Ao et al. and Olusayo et al. screened the preliminary phytochemicals of the EtOH extract of *M. pigra* roots. The tannins, phlobatannins, flavonoids, triterpenes and saponins were found to be present, while alkaloids, anthraquinones and phenolics were found to be absent [94,95]. Rosado-Vallado et al. screened the phytochemicals of the MeOH and aq. extracts of *M. pigra* leaves. The result showed the existence of flavonoids, quinones, saponins, sterols and tannins [96]. Saxena et al. measured the phytochemicals of the EtOH and MeOH extracts of the whole *M. hamata* plant. The preliminary phytochemical analysis indicated the existence of flavonoids, alkaloids, phytosterols, glycosides, tannins, phenolic compounds, saponins and carbohydrates, while proteins and amino acids, fixed oils and fats were found to be absent [97]. Manosroi et al. reported the presence of various phytochemicals, such as flavones, glycosides, saponins alkaloids and tannins, in the aq. extract of *M. invisa* leaves, while anthraquinones and xanthones were found to be absent [98]. Jiménez et al. determined the total phenolic contents of the aq. extract of the whole *M. albida* plant by the Folin–Ciocalteu method. The plant showed a high phenolic content (323 mg GAE/g) [99]. Seraglio et al. measured the phytochemicals of *M. scabrella* bentham honeydew honeys. Coumarins, flavonoids, lignin-derived aldehydes and phenolic acids were found to be present [100]. Nandipati et al. reported on the flavonoids, tannins, triterpenes and carbohydrates in the MeOH extract of the *M. rubicaulis* stem [101]. Table 1 presents various phytochemicals present in different species of the genus *Mimosa.* Phytochemicals are widely known for their medicinal activities. Primary metabolites, such as proteins, lipids and amino acids, are responsible for biochemical reactions in plants, while secondary metabolites, such as saponins, flavonoids, alkaloids, tannins, phenols and glycosides, protect plants against damage and also play a role in the improvement of flavor, color and fragrance. [102]. Phytochemicals in plants are very present in leaves, roots and stems, and their percentage varies because of environmental conditions, plant genus, etc. The *Mimosa* genus is rich in these primary and secondary metabolites, so the plants of this genus are well known for their pharmacological potential.

### 3.2. Bioactive Constitutients of Genus Mimosa

**Figure 1:**
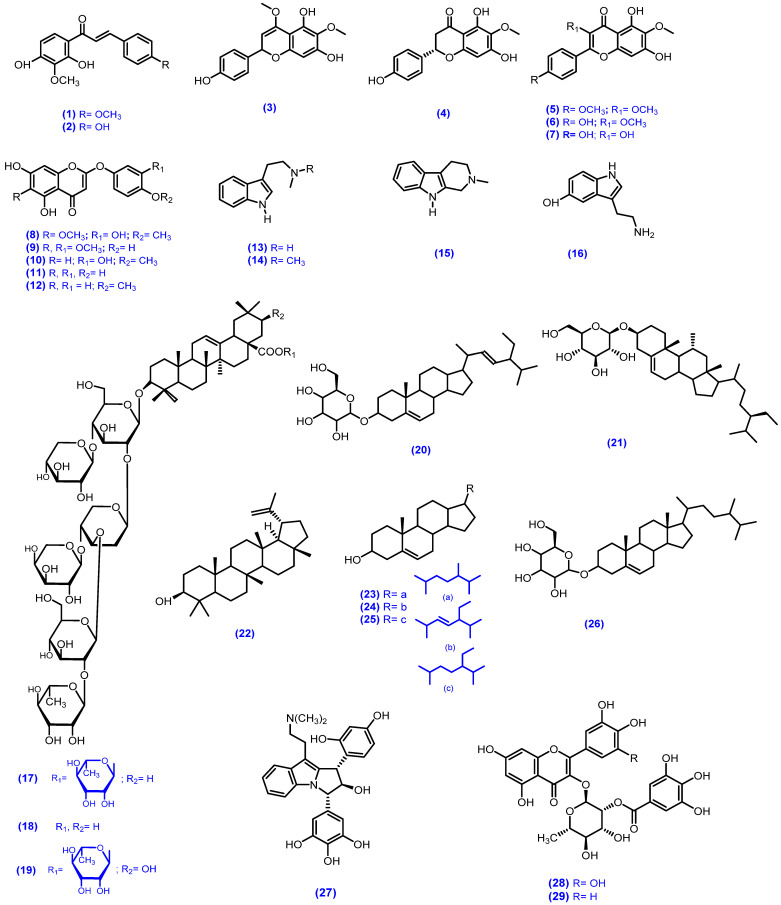

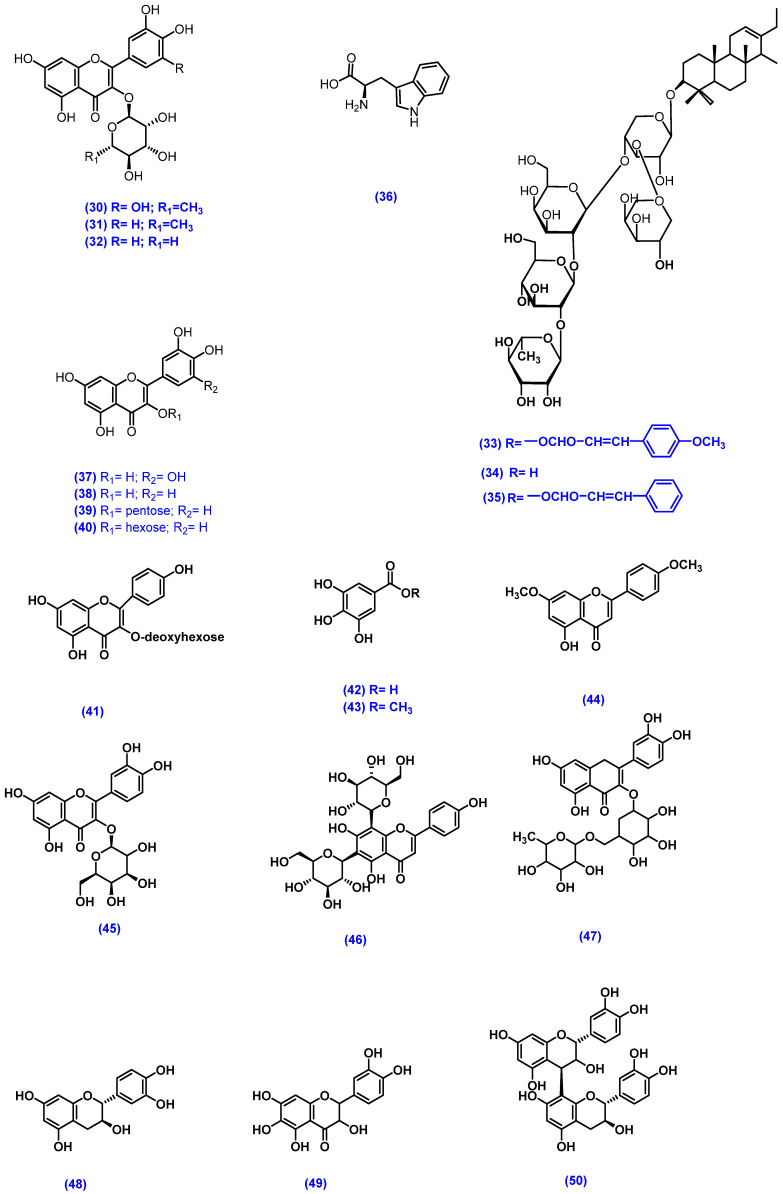

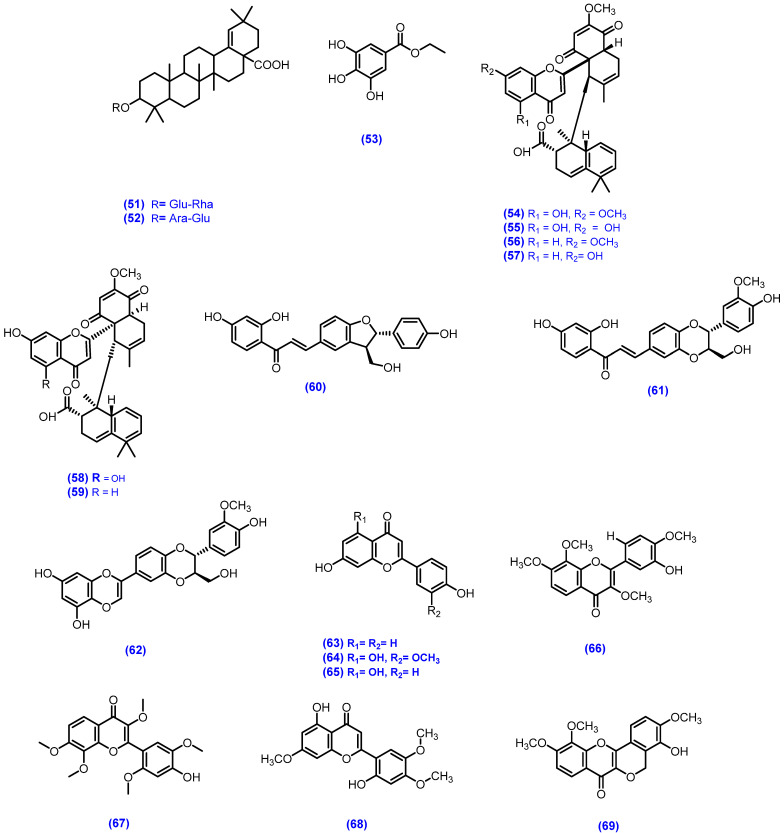

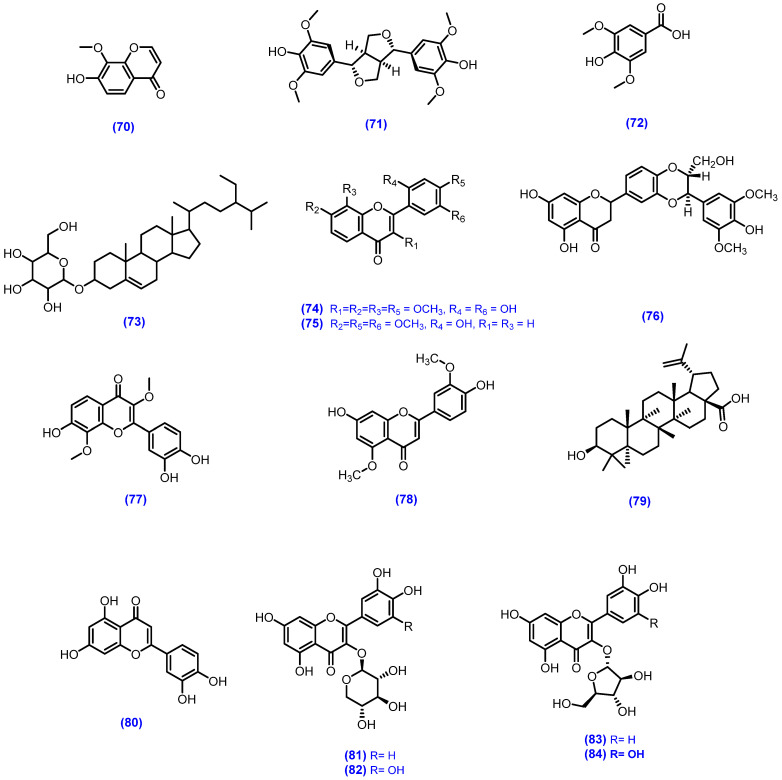

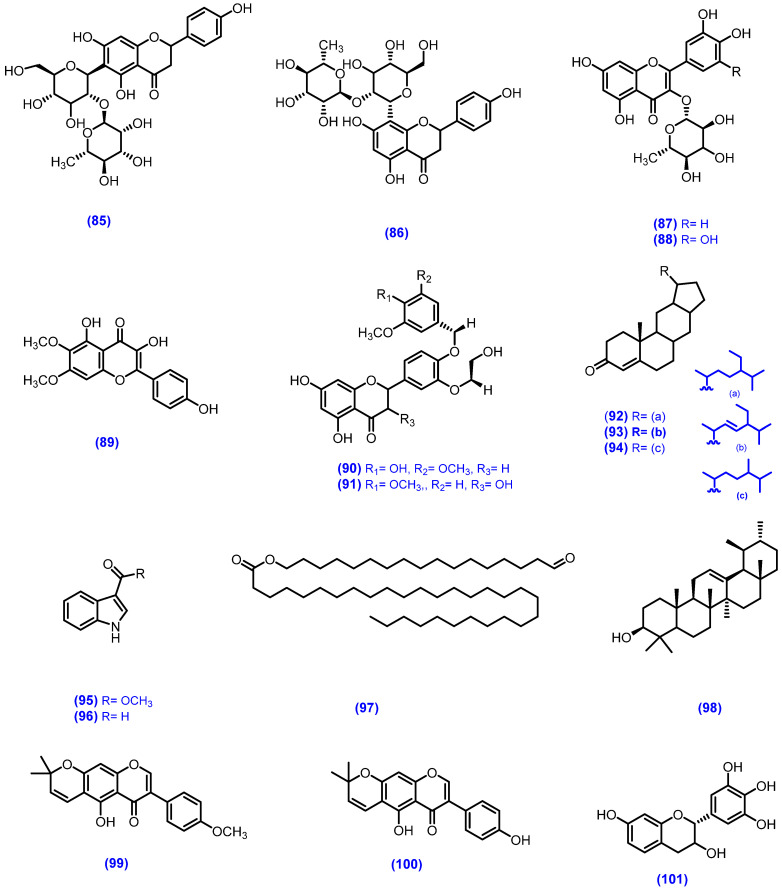

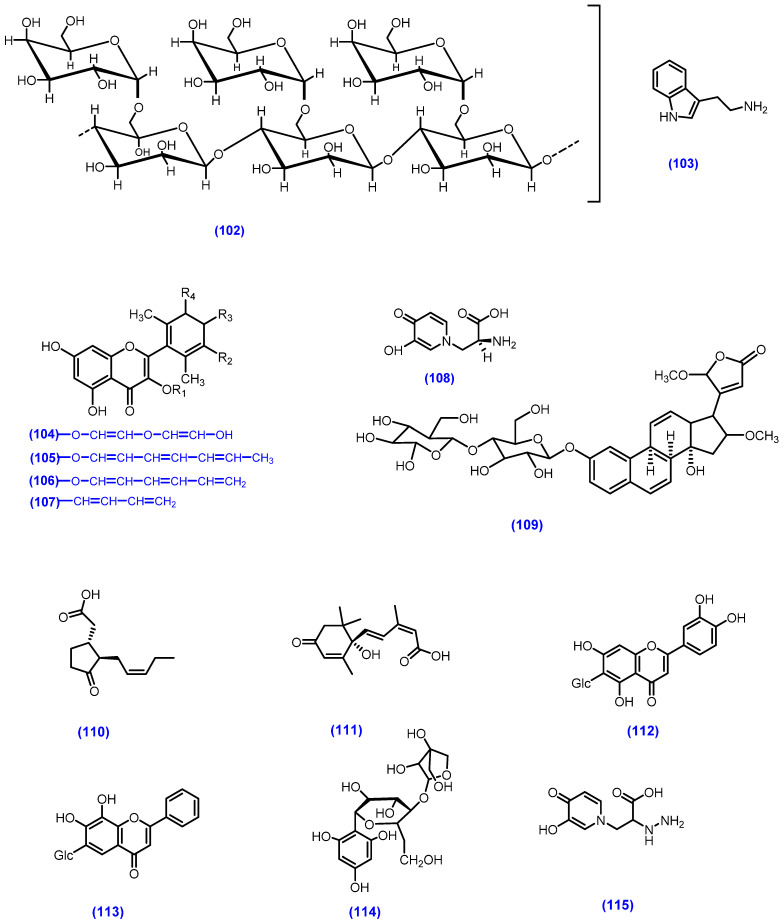

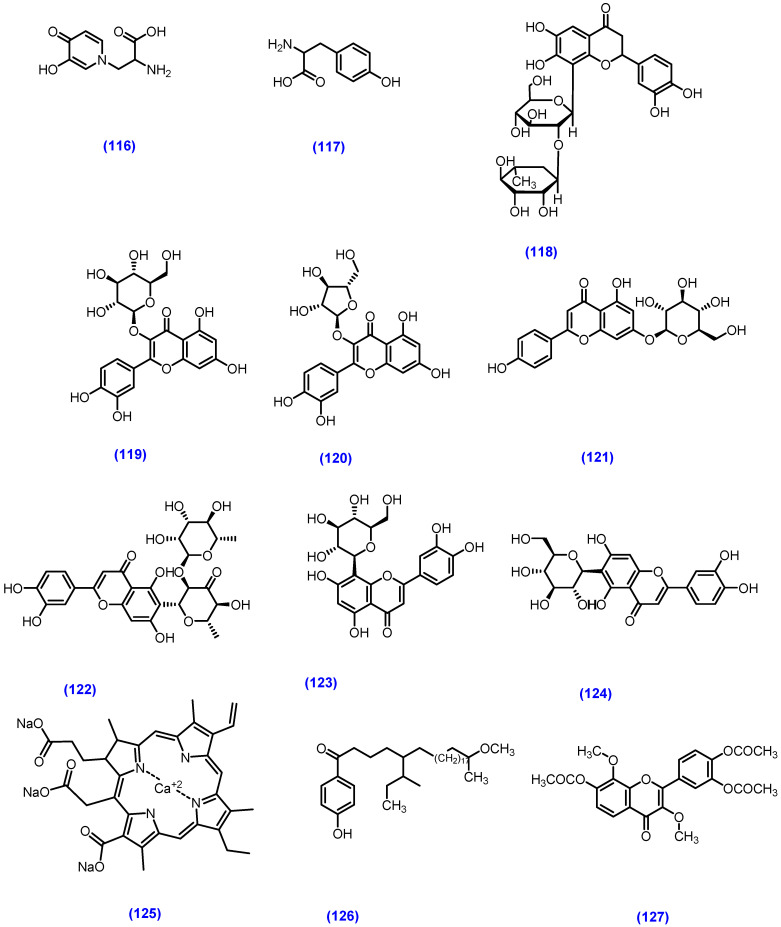

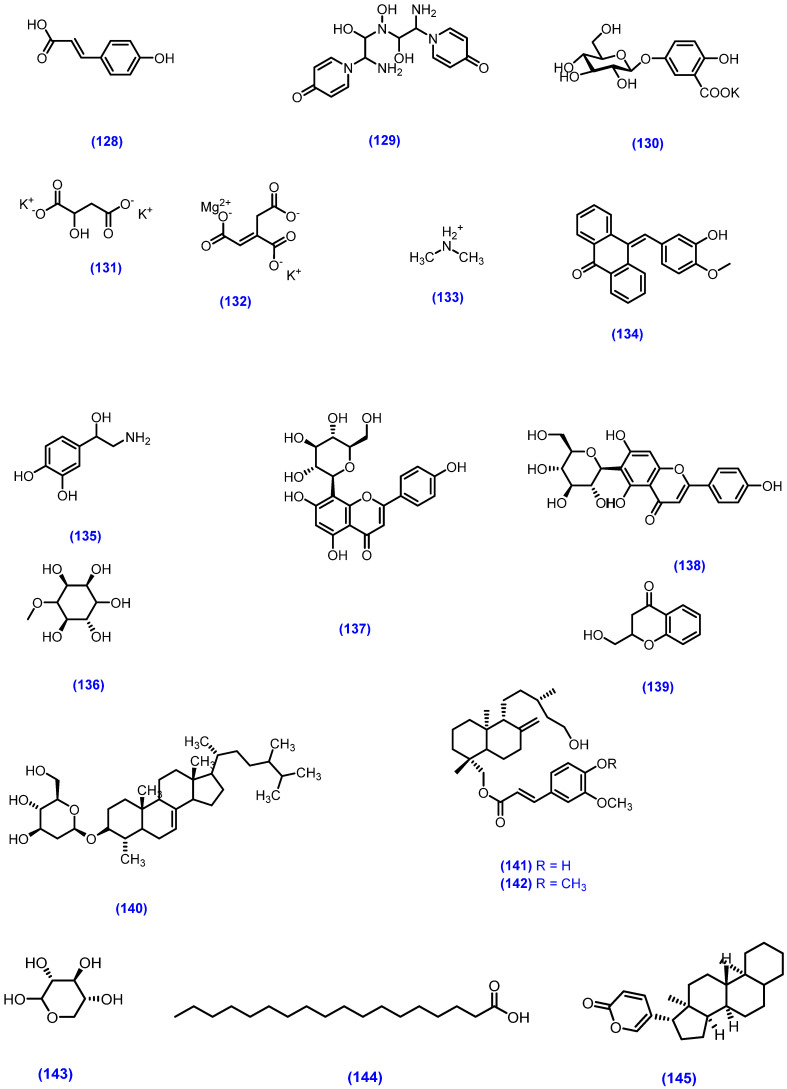
Bioactive constituents isolated from various species of genus *Mimosa*.

The plants of genus *Mimosa* are well known for their rich source of bioactive metabolites. Almost 145 active metabolites have been isolated from the genus *Mimosa* including chalcones, alkaloids, flavonoids, indoles, terpenes, terpenoids, saponins, steroids, amino acids, glycosides, flavanols, phenols, lignoids, polysaccharides, lignins, salts and fatty esters. This part of the paper documents the isolated secondary metabolites of the genus *Mimosa* in the past decades and their pharmaceutical values (Table 2). The structures of all the isolated compounds are presented in Figure 1.

#### 3.2.1. *M. tenuiflora*

Dominguez et al. isolated two chalcones called kukulkan A (2′,4′-dihydroxy-3′,4-dimetoxy chalcone) (**1**) and kukulkan B (2′,4′,4- trihydroxy-3′-metoxychalcone) (**2**) from *M. tenuiflora* stem bark in the form of yellow crystals [103]. Ten different flavonoids including the chromones named 6-methoxy-4-*O*-metylnaringenin (**3**), 6-methoxy naringenin (**4**), santin (**5**), 4,5,7-trihydroxy-3,6-dimethoxy flavone (**6**), 6-methoxykaempferol (**7**), tenuiflorin A [5,7-dihydroxy-2-(3-hydroxy-4-methoxy phenoxy)-6-methoxychromone] (**8**), tenuiflorin B [5,7-dihydroxy-2-(4-hydroxy-3-methoxy phenoxy)-6-methoxychromone] (**9**), tenuiflorin C [5,7-dihydroxy-2-(3-hydroxy-4-methoxy phenoxy)-chromone] (**10**), 6-demethoxycapillarisin (**11**) and 6-demethoxy-4-*O*-methylcapillarisin (**12**) were isolated from the flowers and leaves of *M. tenuiflora* [104,105]. Gardner et al. reported on the alkaloids *N*-methyl-tryptamine (**13**), *N*,*N*-dimethyltryptamine (**14**) and 2-methyltetrahydro-*β*-carboline (**15**) from the MeOH and crude extracts of leaves and seeds of *M. tenuiflora* [106]. Meckes-Lozoya et al. isolated the alkolides 5-hydroxy-tryptamine (**16**) and *N*,*N*-dimethyl tryptamine (**14**) from the Hex:ACE:MeOH extracts of root bark of *M. tenuiflora* [107]. Different terpenoidal saponins called mimonoside A (**17**), mimonoside B (**18**) and mimonoside C (**19**); steroids saponins called stigmasterol-3-*O*-*β*-D-glucopyranosyl (**20**), β-sitosterol-3-*O*-*β*-D-glucopyranosyl (**21**), lupeol (**22**), campesterols (**23**), stigmasterol (**24**), β-sitosterol (**25**) and campesterol-3-*O*-*β*-D-glucopyranosyl (**26**) were isolated from the MeOH extract of the stem bark of *M. tenuiflora* [13,108,109]. The phytoindole alkaloid yuremamine (**27**) was isolated from the MeOH extract of *M. tenuiflora* leaves [110,111].

#### 3.2.2. *M. pigra*

The novel acylated flavanol glycosides myricetin (2-*O*-galloyl)-3-*O*-α-L-rhamnopyranoside (**28**), quercetin(2-*O*-galloyl)-3-*O*-α-L-rhamnopyranoside (**29**), myricetin 3-*O*-α-L-rhamno pyranoside (**30**), quercetin 3-*O*-α-L-rhamnopyranoside (**31**) and quercetin 3-*O*-*β*-L-arabino pyranoside (**32**) were isolated from *M. pigra* leaves [112]. Englert et al. isolated two novel triterpene glycosides called Z/E-methoxycinnamic (**33**,**34**) or an *E*-cinnamic acid (**35**) from the BuOH extract of *M. pigra* stem bark [113]. Rakotomalala et al. reported on the isolation of tryptophan (**36**), myricitrin (**37**), quercitrin (**38**), quercetin 3-*O*-hexose (**39**), quercetin 3-*O*-pentose (**40**) and kampferol 3-*O*-desoxyhexose (**41**) from the hydro-MeOH extract of *M. pigra* leaves [114].

#### 3.2.3. *M. caesalpiniifolia*

Santos et al. isolated gallic acid (**42**), methylgallate (**43**), 5-hydroxy-4,7-dimethoxy-flavone (**44**), quercetin (**38**), quercetin-O-hexoside (**45**), vicenin-2 (**46**) and rutin (**47**) from the EtOH extract of inflorescence of *M. caesalpiniifolia* [115]. Silva et al. isolated the phenolic compounds called catechin (**48**), 2,3 dihydroquercetagetin (**49**) and procyanidin (**50**) from the EtOH extract of *M. caesalpiniifolia* leaves [116].

#### 3.2.4. *M. hamata*

The compounds mimonoside A (**17**), mimonoside B (**18**), mimonoside C (**19**), saponin A (**51**) and saponin B (**52**) were isolated from the leaves and roots of *M. hamata* [117,118,119]. Mehta et al. isolated 4-ethylgallic acid (**53**) from the ACE extract of the flowers of *M. hamata* [60], while gallic acid (**42**) and 4- ethylgallic acid (**53**) were found in the leaves of *M. hamata* [7].

#### 3.2.5. *M. diplotricha*

Chiou et al. isolated six new meroterpenoids called diplomeroterpenoids A-F (**54**–**59**), two new chalcone-lignoids called diplochalcolins A and B (**60**,**61**) and 13 known compounds called hydnocarpin (**62**), 7,4-dihydroxyflavone (**63**), chrysoeriol (**64**), apigenin (**65**), diplotrin B (**66**), 2-hydroxy-3,7,4′,8,5′-pentamethoxyflavone (**67**), hernancorizin (**68**), diplotasin D (**69**), 7-hydroxy-8-methoxychromone (**70**), (+)-syringaresinol (**71**), 4-hydroxy-3,5-dimethoxybenzoic acid (**72**), *β*-sitosterol (**25**), *β*-sitosterol glucoside (**73**) from CHCl_3_ extract of *M. diplotricha* roots [120]. Lin et al. reported four 5-deoxyflavones called diplotrin A (**74**), diplotrin B (**66**), diplotrin C (**75**), diplotasin D (**69**), 5-methoxyhydnocarpin-D (**76**), 7,3′,4′-trihydroxy-3,8-dimethoxyflavone (**77**), 2-hydroxy-3,7,8,4,5-pentamethoxyflavone (**67**), hernancorizin (**68**), 5,3′-di-*O*-methylluteolin (**78**), betulinic acid (**79**), luteolin (**80**), quercetin (**38**), quercetin-3-*O*-xylopyranoside (**81**), myricetin-3-*O*-xylopyranoside (**82**), quercetin-3-*O*-arabino furanoside (**83**) and myricetin-3-*O*-arabino furanoside (**84**) from the EtOH extract of the whole *M. diplotricha* plant [58].

#### 3.2.6. *M. xanthocentra*

Camargo et al. isolated the flavones isovitexin-2-*O*-α-L-rhamnopyranoside (**85**), vitexin-2-*O*-α-L-rhamnopyranoside (**86**), quercetin-3-*O*-xylopyranoside (**81**) and quercetin-3-*O*-arabino furanoside (**83**) from the EtOAc and BuOH fractions of *M. xanthocentra* aerial parts [121].

#### 3.2.7. *M. hostilis*

Pachter et al. isolated indole alkaloid and *N*,*N*-dimethyltryptamine (**14**) from the EtOAc extract of *M. hostilis* roots [122].

#### 3.2.8. *M. artemisiana*

do Nascimento et al. isolated quercetina-3-*O*-raminosídeo (**87**), miricetina-3-*O*-raminoside (**88**), euphaline,3,5,4-trihydroxy-6,7-dimethoxy flavone (**89**), flavonolignana (**90**–**93**), sitosterol-3-*O*-*β*-D glycopyranoside (**21**), lupeol (**22**), steroids sitostenone (**92**), stigmastenone (**93**), campestenone (**94**), campesterol (**23**), stigmasterol (**24**), sitosterol (**25**), methyl indole-3-carboxilate (**95**) and indole-3-carboxaldehyde (**96**) from the *n*-Hex and MeOH extracts of the leaves and branches of *M. artemisiana* [25].

#### 3.2.9. *M. invisa*

Nana et al. isolated new fatty aldol ester called 17-*O*-triacontanoylheptadecanal (**97**) and *β*-sitosterol (**25**), α-amyrine (**98**), lupeol (**22**), 4-*O*-methylepinumisoflavone (**99**), alpinumisoflavone (**100**), betulinic acid (**79**), sitosterol 3-*O*-*β*-D-glucopyranoside (**21**) and epirobinetinidol (**101**) from the aerial parts of the *M. invisa* (DCM/MeOH) extracts [28].

#### 3.2.10. *M. scabrella*

Chrestani et al. isolated polysaccharide and sulfated galactomannan (BRS) (**102**) from the seeds of *M. scabrella* [123].

#### 3.2.11. *M. somniam*

Gupta et al. isolated the alkaloid tryptamine (**103**) and N-methyltryptamine (**13**) from the MeOH extract of the whole *M. somniam* plant [124].

#### 3.2.12. *M. pudica*

##### Whole Plant (Tree) Phytochemicals

Different classes of compounds were isolated from whole *M. pudica* plant (tree). Jose et al. isolated 2-2′,6′-dimethyl-3′,4′,5′-alkyl or hydroxy alkyl substituted phenyl-3-oxy-(alkyl or hydoxy alkyl) 5,7-dihydroxy-chromen-4-one (**104**–**107**) from the EtOAc fraction of *M. pudica* [50]. One amino acid called *L*-mimosine (**108**) was extracted from the hydroalcoholic extract of *M. pudica* [50] [34,125]. Chukwu et al. isolated triterpenoid glycoside (**109**) from the crude EtOH extract of the whole *M. pudica* plant [126]. Tsurumi and Asahi isolated jasmonic acid (**110**) and abscisic acid (**111**) from *M. pudica* [127]. Yuan et al. isolated two new C-glycosyl flavones called 5,7,3′,4′-tetrahydroxy-6-*C*-[*β*-D-apiose-(1→4)]-*β*-D-glucopyranosyl flavones (**112**), 7,8,3′,4′-tetrahy droxyl-6-*C*-[*α*-L-rhamnopyranosyl-(1→2)]-*β*-D-glucopyranosyl flavones (**113**), 5,7,4′-trihydroxyl-8-*C*-*β*-D-glucopyranosyl flavones (**114**), mimosinamine (**115**), mimosinicacid (**116**) and tyrosin (**117**) from the whole *M. pudica* plant. Compound (**112**) is a new compound, and compounds (**113**,**114**) were isolated for the first time from this plant [128]. Yuan et al. also isolated two new C-glycosyl flavones called 5,7,3′,4′-tetrahydroxy-6-*C*-[*β*-D-apiose-(1→4)]-*β*-D-glucopyranosyl flavones (**112**) and 6,7,3′,4′-tetrahydroxyl-8-C-[*α*-L-rhamno pyranosyl-(1→2)]-*β*-D-glucopyranosyl flavone (**118**) from the *M. pudica* plant [129].

##### Aerial Part Phytochemicals

Misra and Tewari isolated six compounds (flavonoids) from the aerial parts of *M. pudica* and identified them as isoquercitrin (**119**), avicularin (**120**), apigenin-7-*O*-D-glucoside (**121**), cassiaoccidentalin B (**122**), orientin (**123**) and isoorientin (**124**) [130].

##### Leaf Phytochemicals

Rajalakshmi and Banu reported the presence of chlorophyllin (**125**) in the MeOH extract of fresh *M. pudica* leaves [131]. Josewin et al. isolated the phenolic ketone called 4-(24′-methoxy-24′-methyl-1′-oxo-5′-n-propyl-tetracosanyl)- phenol (**126**) from the leaves of *M. pudica* [132]. Kirk et al. isolated three flavonoids called 7, 3′,4′-Triacetoxy-3,8-dimethoxyflavone (**127**), *p*-coumaric acid (**128**) and 7,3′,4′-trihydroxy-3,8-dimethoxyflavone (**77**) from the leaves of *M. pudica* [133]. Ueda and Yamamura isolated a leaf opening compound called mimopudine (**129**) and a leaf closing compound called potassium 5-*O-β*-D-glucupyranosylgentisate (**130**) from the leaves of *M. pudica.* This compound mimopudine (**129**) is responsible for the opening and movements of leaves even at night, while the compound mimopudine (**130**) is responsible for the closing of the leaves [134,135]. Ueda and Yamamura also isolated different chemical substance such as mimopudine (**129**), glucupyrano sylgentisate (**130**), potassium L-malate (**131**), magnesium potassium trans-aconitate (**132**) and dimethyl ammonium salt (**133**) from *M. pudica* leaves. These compounds are responsible for rapid sensitive actions, such as heat and touch, and episodic slow actions, such as nyctinastic actions [136]. Pal et al. isolated tubulin protein (134) (pulvinar callus cells) from the fresh leaves of *M. pudica* [137]. Khare isolated three compounds as nor-epinephrine (**135**), d-pinitol (**136**) and β-sitosterol (**25**) from *M. pudica* leaves [138]. Zhang et al. isolated five flavonoids named as 5,7,3′,4′tetrahydroxy-6-C-[β-D-apiose-(1→4)]-β-D-glycopyranosyl flavone (112), orientin (123), isorientin (124), vitexin (137) and isovitexin (138) from *M. pudica* leaves [139].

##### Root Phytochemicals

Kanga et al. reported a new chroman called 2-hydroxymethyl-chroman-4-one (**139**) from *M. pudica* roots [140]. Dinda et al. isolated a new sterolglucoside called 4-a,24-dimethylcholest-7-en-3*β*-ol-3*β*-D-glucoside (**140**) along with three other compounds called stigmasterol (**24**), β-sitosterol (**25**) and betulinic acid (**79**) from the roots of *M. pudica* [141]. Shu and Ho (2013) isolated two new diterpenoids named 19-*O*-trans-feruloyl-labd-8(17)-en-15,19-diol (**141**) and 19-*O*-[(*E*)-3′,4′-dimethoxy cinnamoyl]-labd-8(17)-en-15,19-diol (**142**) from the roots of *M. pudica* [142].

##### Seed Phytochemicals

Chatterjee and Pakrashi isolated two compounds called D-xylose (**143**) and D-glucuronic acid 4-*O*-(3,5-dihydroxybenzoic acid)-β-D-glucuronide (**144**) in the form of mucilage from *M. pudica* seeds [143]. Yadava and Yadav reported a novel compound bufadienolide (hellebrigenin-3-O-α-l-rhamnopyranosyl-(1→4)-O-β-D-galactopyranoside) (**145**) from the seeds of *M. pudica* [144].

##### Stem Phytochemicals

Zaware et al. also isolated nonprotein amino acid called mimosine; [β-[N-(3-hydroxy-4-oxypyridyl)]-α-aminopropionic acid] (**108**) from the stem of *M. pudica* [145].

## 4. Pharmacological Activities of Genus *Mimosa*

The potential role of the *Mimosa* genus in traditional medicines encouraged the further biological evaluations of organic extracts and isolated phytoconstituents for potential pharmacological applications. In this section, we summarized the pharmacological activities of genus *Mimosa* (Table 2).

### 4.1. Antiprotozoal Activity

Antiprotozoals are drugs that are used to treat different infections including babesiosis, microsporidiosis, amebiasis, malaria, leishmaniasis and malaria. These infections are caused by various protozoa. Currently, the treatments of these infections are limited because of toxicity. So, there is a need to find new natural sources to treat these infections with less toxicity. A group of scientists determined the antiprotozoal activity of ten different flavonoids and chromones including 6-methoxy-4-*O*-metylnaringenin (**3**), 6-methoxy naringenin (**4**), santin (**5**), 4,5,7-trihydroxy-3,6-dimethoxy flavone (**6**), 6-methoxykaempferol (**7**), tenuiflorin A (**8**), tenuiflorin B (**9**), tenuiflorin C (**10**), 6-demethoxycapillarisin (**11**) and 6-demethoxy-4-*O*-methyl capillarisin (**12**) isolated from the leaves and flowers of *M. tenuiflora* against *E. histolytica and G. lamblia.* The most interesting activity was obtained with (**8**) (IC_50_ = 41.1 μg/mL) against *E. histolytica* and (**5**) (IC_50_ = 75.3 μg/mL) against *G. lamblia* [104,105].

### 4.2. Antimicrobial Activity

Jain et al. observed the antimicrobial activity of mimonoside A (**17**), mimonoside B (**18**), mimonoside C (**19**), saponin A (**51**) and saponin B (**52**) isolated from the leaves and roots of *M. hamata* against different biological strains, such as *E. coli, B. subtilis, S. aureus, P. aeruginosa, A. flavus*, *E. aerogenes*, *K. pneumoniae*, *A. niger*, *C. albicans* and *R. bataticola,* using the agar well diffusion method. All compounds showed significant activity against Gram-negative bacteria and fungi, while moderate activity was observed against Gram- positive bacteria compared to standard gentamycin (10 μg/mL) and ketoconazole (100 units/mL). None of the saponins was active against *A. niger*, *A. flavus* and *C. albicans* [119]. Nana et al. measured the antimicrobial activity of new fatty aldol ester called 17-*O*-triacontanoylheptadecanal (**97**) and *β*-sitosterol (**25**), *α*-amyrine (**98**), lupeol (**22**), 4-*O*-methylepinumisoflavone (**99**), alpinumisoflavone (**100**), betulinic acid (**79**), sitosterol 3-*O*-*β*-D-glucopyranoside (**21**) and epirobinetinidol (**101**) from the aerial parts of *M. invisa* against *E. coli, E. aerogenes, S. aureus, P. aeruginosa, K. pneumoniae, S. typhi and C. albicans* using the XTT colorimetric assay. Compound (**97**) displayed antimicrobial activity with MIC values ranging from 64 µg/mL to 256 µg/mL. Both compounds showed pronounced activity against *K. pneumoniae* (MIC = 64 µg/mL). The antimicrobial activity of chlorophyllin (**125**) extracted from the fresh leaves of *M. pudica* against *P. aeruginosa*, *E. coli*, *S. aureus*, *K. pneomoniae*, *C. albicans* was determined by using the agar well diffusion method [131]. The compound (**125**) at a concentration of 25 µg/mL showed a significant zone of inhibition against *P. aeruginosa = 12 mm*, *E. coli = 8 mm*, *S. aureus = 14 mm*, *K. pneumoniae = 13 mm* and *C. albicans = 9 mm*, while at a concentration of 100 µg/mL, *P. aeruginosa*, *E. coli*, *S. aureus*, *K. pneumoniae* and *C. albicans* = 18 mm, 13 mm, 19 mm and 18 mm inhibition was observed, respectively. The streptomycin sulphate and nystatin (standard) at 10 µg/mL showed a maximum inhibition of 18 mm–19 mm [28]. The antifungal activity of 2-hydroxymethyl-chroman-4-one (**139**) isolated from *M. pudica* roots was checked against various strains, such as *P. ultimum*, *P. capsici*, *R. solani*, *B. cinerea*, *A. panax* and *S. sclerotiorum*, by using the dilution agar plate method. Compound (**139**) showed a significant ED_50_ value against *P. capsici*, *S. sclerotiorum* and *P. ultimum =* 5.7 µg/mL, 52.1 µg/mL and 54.9 µg/mL, respectively [140].

### 4.3. Antiviral Activity

Chrestani et al. measured the antiviral activity of the polysaccharide sulfated galactomannan (BRS) (**102**) isolated from the seeds of *M. scabrella* to counter the *Herpes simplex* virus 1 (HSV-1) at a concentration range of 2 µg/mL–2.5 µg/mL. The (**102**) exhibited IC_50_ value was less than 2.5 µg/mL. Even at a very low concentration (2.5 µg/mL), viral activity was inhibited [123].

### 4.4. Antioxidant Activity

The reactive oxygen species are the cause of many diseases in human beings including cardiovascular and neurological disorders [146]. There is a constant need to counteract the effect of reactive oxidative species to delay the progression on these diseases. Reactive oxygen species and free radicals are scavenged by antioxidants through the termination of a chain reaction, which otherwise can cause damage to cells [147]. Plants are a source of antioxidants and can help to lessen the diseases caused by reactive oxidative species. Natural antioxidants are more potent and less toxic, so there is a need to find more natural sources for well-being. Various species of *Mimosa* have been screened for antioxidant activity by the application of different assays (Table 2). Jain et al. measured the antioxidant activity of mimonoside A (**17**), mimonoside B (**18**), mimonoside C (**19**), saponin A (**51**) and saponin B (**52**) isolated from the leaves and roots of *M. hamata* using the DPPH free radical scavenging assay. Compounds revealed significant IC_50_ values; (**17**) = 0.45 µg/mL; (**18**) = 0.55 µg/mL; (**19**) = 0.60 µg/mL; (**51**) = 0.085 µg/mL and (**52**) = 0.10 µg/mL, while quercetin (Standard) showed an IC_50_ value of 0.06 µg/mL [119]. The amino acid *L*-mimosine (**108**) was extracted from the hydroalcoholic extract of *M. pudica,* and the antioxidant activity was determined by the DPPH radical scavenging assay. The compound (**108**) at a concentration of 250 μM showed significant activity (IC_50_ = 233.06 μM) [34,125].

### 4.5. Antiproliferative Activity

Chiou et al. investigated the antiproliferative activity of isolated compounds called diplomero terpenoids A–F (**54**–**59**), two new chalcone-lignoids called diplochalcolins A and B (**60**,**61**) and 13 known compounds called hydnocarpin (**62**), 7,4-dihydroxyflavone (**63**), chrysoeriol (**64**), apigenin (**65**), diplotrin B (**66**), 2-hydroxy-3,7,4′,8,5′-pentamethoxyflavone (**67**), hernancorizin (**68**), diplotasin D (**69**), 7-hydroxy-8-methoxychromone (**70**), (+)-syringaresinol (**71**), 4-hydroxy-3,5-dimethoxybenzoic acid (**72**), *β*-sitosterol (**25**) and *β*-sitosterol glucoside (**73**) isolated from *M. diplotricha* roots against human hepatoblastoma HepG_2_ cells by the Sulforhodamine B assay (SRB). Compound **54** displayed growth inhibition activity with GI_50_ = 8.6 µM, although the GI_50_ values of the compounds (**55−60**) were greater than 10 Μm [120].

### 4.6. Cytotoxic Activity

Cancer is an uncontrolled and abnormal symmetric growth of body cells [148]. Different cancers were observed in humans, such as in the liver, stomach, lung, breast, prostate, thyroid and cervix. Bioactive compounds isolated from plants are used in curing cancer because they are inexpensive, nontoxic and easily available as compared to synthetic compounds. The bioactive compounds in plants induced apoptosis in infective cancer cells and also helped to restore chemotherapy sensitivity [149]. Lin et al. reported the cytotoxic activity of four 5-deoxyflavones called diplotrin A (**74**), diplotrin B (**66**), diplotrin C (**75**), diplotasin D (**69**), 5-methoxyhydnocarpin-D (**76**), 7,3′,4′-trihydroxy-3,8-dimethoxyflavone (**77**), 2-hydroxy-3,7,8,4,5 pentamethoxy flavone (**67**), hernancorizin (**68**), 5,3′-di-*O*-methylluteolin (**78**), betulinic acid (**79**), luteolin (**80**), quercetin (**38**), quercetin-3-*O*-xylopyranoside (**81**), myricetin-3-*O*-xylopyranoside (**82**), quercetin-3-*O*-arabino furanoside (**83**) and myricetin-3-*O*-arabino furanoside (**84**) isolated from the whole *M. diplotricha* plant against the A549, HT-29, AGS and PC_3_ human cancer cell lines using the SRB assay. Compounds (**66**) and (**76**) presented the powerful antiproliferative activity with GI_50_ values of (**66**) = 2.7 μM, 1.7 μM, 7.5 μM, and 20.8 μM, respectively and (**76**) 20.3 μM, 24.8 μM, 4.1 μM, and 2.3 μM, respectively toward the four human cancer cell lines, while all the other compounds were found >10 Μm [58]. Chrestani et al. measured the cytotoxic potential of sulfated galactomannan (BRS) (**102**) isolated from the seeds of *M. scabrell* against the Vero and MA-104 human cell lines by the MTT assay. At a concentration of ≥ 39 µg/mL, BRS reduced by 15% the viability of Vero cells (CC_50_ = 454 µg/mL), while at a concentration of 625 µg/mL, BRS reduced by 24% the viability of MA-104 [123]. Scientists screened the cytotoxicity of the isolated (2-(2′,6′-dimethyl-3′,4′,5′-alkyl or hydroxy alkyl substituted phenyl)-3-oxy-(alkyl or hydoxy alkyl) 5,7-dihydroxy-chromen-4-one) (**104**–**107**) from *M. pudica* using the MTT assay against human lung adenocarcinoma (A549) and the erythroleukemic cell line (K562). The compound showed significant IC_50_ against A549 = 76.67 µg/mL and K562 = 287.63 µg/mL, while the positive control Doxorubicin showed an IC_50_ value of 2.76 µg/mL and K562 = 4.72 µg/mL against A549 [50]. The cytotoxic potential of the amino acid *L*-Mimosine (**108**) extracted from *M. pudica* by the MTT assay against the daudi cell line was reported. After 72 h, the compound (**108**) showed an IC_50_ value of 86.61 μM. Compound (**108**) act as powerful inhibitors of cell proliferation and showed remarkable cytotoxic activity [34,125].

## 5. Marker Compounds of Genus *Mimosa*

Some of the identified compounds from genus *Mimosa* are specific marker components of this genus. Two chalcones called kukulkan A (**1**) and kukulkan B (**2**) [103] and different terpenoidal saponins called mimonoside A (**17**), mimonoside B (**18**) and mimonoside C (**19**) [13,108,109] are specifically identified in M. tenuiflora. Six new meroterpenoids called diplomero terpenoids A-F (**54**–**59**), two new chalcone-lignoids called diplochalcolins A and B (**60**,**61**) have been identified in *M. diplotricha* roots [120]. Similarly, four 5-deoxyflavones called diplotrin A (**74**), diplotrin B (**66**), diplotrin C (**75**) and diplotasin D (**69**) were specifically found in the whole *M. diplotricha* plant [58]. One amino acid called *L*-mimosine (**108**) was extracted from the hydroalcoholic extract of *M. pudica* [50]. Novel C-glycosyl flavones called 5,7,3′,4′-tetrahydroxy-6-*C*-[*β*-D-apiose-(1→4)]-*β*-D-glucopyranosyl flavones (**112**), 7,8,3′,4′-tetrahy droxyl-6-*C*-[*α*-L-rhamnopyranosyl-(1→2)]-*β*-D-glucopyranosyl flavones (**113**), 5,7,4′-trihydroxyl-8-*C*-*β*-D-glucopyranosyl flavones (**114**), mimosinamine (**115**) and mosinicacid (**116**) were isolated from the whole *M. pudica* plant [128]. A leaf opening compound called mimopudine (**129**) and a leaf closing compound called potassium 5-*O-β*-D-glucupyranosylgentisate (**130**) were isolated from the leaves of *M. pudica.* This compound called mimopudine (**129**) is responsible for the opening and movements of leaves even at night, while the compound mimopudine (**130**) is responsible for the closing of leaves [134,135]. Similarly, potassium L-malate (**131**), magnesium potassium trans-aconitate (**132**) and dimethylammonium salt (**133**) from *M. pudica* leaves were isolated, and these compounds were responsible for rapid sensitive actions, such as heat and touch, and episodic slow actions, such as nyctinastic actions [136]. A new chroman called 2-hydroxymethyl-chroman-4-one (**139**) [140] and a new sterolglucoside 4-a,24-dimethylcholest-7-en-3*β*-ol-3*β*-D-glucoside (**140**) were specifically identified from the roots of *M. pudica* [141]. Two new diterpenoids named 19-*O*-trans-feruloyl-labd-8(17)-en-15,19-diol (**141**) and 19-*O*-[(*E*)-3′,4′-dimethoxy cinnamoyl]-labd-8(17)-en-15,19-diol (**142**) were isolated from the roots of *M. pudica* [142]. A novel compound called bufadienolide (hellebrigenin-3-O-α-l-rhamnopyranosyl-(1→4)-O-β-D-galactopyranoside) (**145**) was specifically isolated from the seeds of *M. pudica* [144].

## 6. Conclusions and Future Perspectives

This review summarized the isolated phytochemical and pharmacological characteristics of the *Mimosa* genus. Out of 400 species only 25 have been chemically studied, while compounds belonging to different chemical classes have been isolated in the *Mimosa* species, such as alkaloids, chalcones, flavonoids, indoles, terpenes, terpenoids, saponins, steroids, amino acids, glycosides, flavanols, phenols, lignoids, polysaccharides, lignins and fatty esters. Significant bioactivities, such as antimicrobial, cytotoxic, antioxidant, antiprotozoal, antiviral and antiproliferative, were discussed in this review. In this review, *M. pudica* was the most studied specie. This review also covered the qualitative and quantitative analysis of phytochemicals, such as flavonoids, steroids, saponins, alkaloids, coumarins, tannins and terpenoids, in the genus *Mimosa*. This review focused on the medicinal aspects of the *Mimosa* species and may provide a comprehensive understanding of the prospective of this genus as a foundation of medicine, supplement and nourishment. Several studies were performed to establish the pharmacological potential by identifying the bioactive secondary metabolites associated with the respective activities. Many studies have been carried out that show this genus possesses huge potential for new drug sources, but there are still gaps, which are noteworthy. Few species of this genus have been explored, so there is a need to explore all species of this genus to find their potential medicinal values for well-being. Secondly, there is a need to provide detailed mechanistic studies on the pharmacology to provide a good understanding of the application of the *Mimosa* species as a source of potential medicines. Thirdly, further studies are required to explore safety aspects of the diverse range of the *Mimosa* species, including chronic toxicity with a determination of the molecular pathways of the health-promoting features of this genus, and attempts are required to isolate more bioactive compounds of this genus.

## Figures and Tables

**Table 1:** Phytochemical screening of different species of genus *Mimosa*.

Plant	Plant Part/Extract	Phytochemicals	References
*M. tenuiflora*	Whole plant	condensed tannins, procyanidin, prodelphinidins	[82]
	leaves, stem	total phenols, total tannins and condensed tannins	[83]
*M. pudica*	Leaves/EtOH extract	alkaloids, flavonois, saponins and triterpenes	[84]
	Whole plant/EtOH extract	alkaloids, flavonoids, steroids, saponins, phenols, tannins, cyanogenic glycosides and anthocyanins	[85]
	Leaves/aq. extract	flavonoids, alkaloids, proteins, steroids, tannins, saponins and terpenoids	[24]
	Leaves/MeOH extract	alkaloids, glycosides, terpenoids and amino acids	[86]
	Aerial parts	alkaloids, saponins, flavonoids, terpenoids and coumarins	[29]
	Roots/EtOH extract	proteins, sterols, tannins, terpenoids, alkaloids, flavonoids and phenolic compounds	[34]
	Whole plant/aq. extract	alkaloids, flavonoids and tannins	[87]
	Leaves and roots/EtOH, MeOH, PE and ACE extracts	flavonoids, alkaloids, terpenoids, carbohydrates, saponins, amino acids, phenols, tannins, proteins and steroids	[88]
	Leaves/ACE, EtOH and aq. extracts	tannin, terpenoids, phlobatannins, steroids, saponin and glycoside	[89]
	Whole plant/MeOH extract	flavonoids alkaloids, saponins, terpenoids, phenols, glycosides, tannins, and coumarins	[26]
	Leaves and stem/aq. extract	saponins, alkaloids, flavonoids, tannins and phenols	[90,91]
	Leaves and stem/aq. extract	magnesium, phosphorus, calcium, nitrogen and potassium	[91]
	Leaves	neoxanthin, viola xanthin, lutein, lycopene, carotenes, tocopherol, total carotenoids and total vitamins	[92]
	Whole plant/EtOH extrac	alkaloids, flavonoids, tannins, phenolics and steroids	[93]
*M. pigra*	Roots/EtOH extract	tannins, phlobatannins, flavonoids, triterpenes and saponins	[94,95]
	Leaves/MeOH and aq. extracts	flavonoids, quinones, saponins, sterols and tannins	[96]
*M. hamata*	Whole plant/EtOH and MeOH extracts	flavonoids, alkaloids, phytosterols, glycosides, tannins, phenolic compounds, saponins and carbohydrates,	[97]
*M. invisa*	Leaves/aq. extract	flavones, glycosides, saponins alkaloids and tannins	[98]
*M. albida*	Whole plant/aq. extract	total phenolic contents	[99]
*M. scabrella bentham*	Honeydew honeys	lignin-derived aldehydes, coumarins, phenolic acids and flavonoids	[100]
*M. rubicaulis*	Stem/MeOH extract	flavonoids, tannins, triterpenes and carbohydrates	[101]

**Table 2:** Bioactive metabolites of genus *Mimosa* and their pharmacological potential.

Species	Extract	Parts	Classification	Compounds	Modal/Assay	Responses along with Critical Assessment	Ref.
*M. tenuiflora*	DCM-Hex -MeOH	Stem bark	Chalcones	kukulkan A (**1**), kukulkan B (**2**)			[103]
	Hex, ACE, MeOH	Leaves and flowers	Flavonoids	6-methoxy-4-*O*-metylnaringenin (**3**), 6-methoxynaringenin (**4**), santin (**5**), 4,5,7-trihydroxy-3,6-dimethoxy flavone (**6**), 6-methoxykaempferol (**7**), tenuiflorin A (**8**), tenuiflorin B (**9**), tenuiflorin C (**10**), 6-demethoxycapillarisin (**11**), 6-demethoxy-4-*O*-methyl capillarisin (**12**)	In vitro/Antiprotozoal assays/*E. histolytica, G. lamblia*	(IC_50_ μg/mL) against *E. histolytica* and *G. lamblia* (**3**) = 72.7 μg/mL and 82.9 μg/mL; (**4**) = 69.7 μg/mL and 75.3 μg/mL; (**5**) = 76.4 μg/mL and 84.1 μg/mL; (**6**) = 41.1 μg/mL and 108.6 μg/mL; (**7**) = 69.8 μg/mL and 77.1 μg/mL; (**8**) = 80.7 μg/mL and 91.8 μg/mL; (**9**) = 71.6 μg/mL and 77.8 μg/mL; (**10**) = 82.8 μg/mL and 92.8 μg/mL; (**11**) = 89.9 μg/mL and 100.9 μg/mL; (**12**) = 78.7 μg/mL and 86.6 μg/mL; positive control; emetinec = 2.2 μg/mL and 0.8 μg/mL; Metronidazolec = 0.23 μg/mL and 1.22 μg/mL respectively. Overall good activity	[104,105]
	MeOH, Crude Alkaloid Extracts	Leaves and seeds	Indole alkaloid	*N*-methyltryptamine (**13**), *N*,*N*-dimethyltryptamine (**14**), 2-methyltetrahydro-*β*-carboline (**15**)			[106]
	Hex; ACE: MeOH	Trunk bark, root bark	Indole alkaloid	*N*, *N*-dimethyltryptamine (**14**), 5-hydroxy-tryptamine (**16**)			[107]
	MeOH	Stem bark	Terpenoids saponins	mimonoside A (**17**), mimonoside B (**18**), mimonoside C (**19**)			[13,108,109]
	MeOH	Stem bark	Steroids saponins	stigmasterol-3-*O*-*β*-D-glucopyranosyl (**20**), *β*-sitosterol 3-*O*-*β*-D-glucopyranosyl (**21**), lupeol (**22**), campesterols (**23**), stigmasterol (**24**), β-sitosterol (**25**), campesterol-3-*O*-β-D-glucopyranosyl (**26**)			[108]
	MeOH	Root bark, stem bark	*phytoindole* Alkaloid	yuremamine (**27**)			[110,111]
*M. pigra*	DEE and EtOAc fraction	Leaves	Acylated flavonol glycosides	myricetin (2-*O*-galloyl)- 3-*O*-*α*-L-rhamnopyranoside (**28**), quercetin (2-*O*-galloyl)- 3-*O*-*α*-L-rhamnopyranoside (**29**), myricetin 3-*O*-*α*-L-rhamnopyranoside (**30**), quercetin 3-*O*-*α*-L-rhamnopyranoside (**31**), quercetin 3-*O*-*β*-L-arabinopyranoside (**32**)			[112]
	BuOH	Stem bark	Triterpene glycosides	Z/E-methoxycinnamic (**33**,**34**), E-cinnamic acid (35)			[113]
	Hydro-MeOH extract	Leaves	Tryptophan, amino acid and phenols	tryptophan (**36**), myricitrin (**37**), quercitrin (**38**), quercetin 3-*O*-pentose (**39**), quercetin 3-*O*-hexose (**40**), kampferol 3-*O*-desoxyhexose (**41**)			[114]
*M. caesalpiniifolia*	EtOH extract	Inflorescence		gallic acid (**42**), methylgallate (**43**), 5-hydroxy-4,7-dimethoxy-flavone (**44**), quercetin (38), quercetin-*O*-hexoside (**45**), vicenin-2 (**46**), rutin (**47**)			[115]
	EtOH extract	leaves	Phenols and flavonids	catechin (**48**), 2,3 dihydroquercetagetin (**49**), procyanidin (**50**)			[116]
*M. hamata*	MeOH extract	Roots	Triterpenoidal Saponins	mimonoside A (**17**), mimonoside B (**18**), mimonoside C (**19**), saponin A; (3-*O*-L rhamnopyran osyl -D-glucopyranosylmorolic acid) (**51**), saponin B; (3-*O*-L-Arabinosyl-D-glucosylmorolic acid) (**52**)	In vitro/Antimicrobial activity agar well diffusion method/against *B. subtilis, E. coli, E. aerogenes*, *S. aureus, P. aeruginosa, A. flavus*, *K. pneumonia*, *A. niger*, *C. albicans, R. bataticola*	Good activity against Gram -ve bacteria and fungi Marginal activity toward Gram +ve bacteria	[117,118,119]
					Antioxidant activity/DPPH free radical scavenging assay	Compounds exhibited IC_50_; (**17**) = 0.45 μg/mL; (**18**) = 0.55 μg/mL; (**19**) = 0.60 μg/mL; (**51**) = 0.085 μg/mL; (**52**) = 0.10 μg/mL; Standard Quercetin = 0.06 μg/mL. Overall good activity	[119]
	ACE	Flowers, leaves		4-ethylgallic acid (**53**), gallic acid (**42**)			[7,60]
*M. diplotricha*	CHCl_3_	Root	Meroterpenoids, chalcone-lignoids	diplomeroterpenoid A (**54**), diplomeroterpenoid B (**55**), diplomeroterpenoid C (**56**), diplomeroterpenoid D (**57**), diplomeroterpenoid E (**58**), diplomeroterpenoid F (**59**), diploflavolin A (**60**), diploflavolin B (**61**), hydnocarpin (**62**), 7,4-dihydroxyflavone (**63**), chrysoeriol (**64**), apigenin (**65**), diplotrin B (**66**), 2-hydroxy-3,7,4′,8,5′-pentamethoxyflavone (**67**), hernancorizin (**68**), diplotasin D (**69**), 7-hydroxy-8-methoxychromone (**70**), (+)-syringaresinol (**71**), 4-hydroxy-3,5-dimethoxybenzoic acid (**72**), *β*-sitosterol (**25**), *β*-sitosterol glucoside (**73**)	Antiproliferative activity against human hepatoblastoma HepG2 cells/SRB assay	Compound **54** showed antiproliferative activity GI_50_ = 8.6 μM) while Compounds **55**–**60** ≥ 10 μM. Marginal activity	[120]
	EtOH	Whole plant	5-deoxy flavones, flavonoids, flavonolignans and triterpenoids	diplotrin A (**74**), diplotrin B (**66**), diplotrin C (**75**), diplotasin D (**69**), 5-methoxyhydnocarpin-D (**76**), 7,3′,4′-trihydroxy-3,8-dimethoxyflavone (**77**), 2-hydroxy-3,7,8,4,5 pentamethoxy flavone (**67**), hernancorizin (**68**), 5,3′-di-*O*-methylluteolin (**78**), betulinic acid (**79**), luteolin (**80**), quercetin (**38**), quercetin-3-*O*-xylopyranoside (**81**), myricetin-3-*O*-xylopyranoside (**82**), quercetin-3-*O*-arabino furanoside (**83**), myricetin-3-*O*-arabino furanoside (**84**)	In vitro/Cytotoxic activity/A549, AGS, HT-29, and PC3 human cancer cell line/SRB assay	Against all cell lines GI_50_ (**66**) = 2.7 μM, 1.7 μM, 7.5 μM, and 20.8 μM, (**76**) 20.3 μM, 24.8 μM, 4.1 μM, and 2.3 μM, respectively. Excellent activity.	[58]
*M. xanthocentra*	EtOAc, BuOH	Aerial parts	flavones	isovitexin-2-*O*- α rhamnopyranoside (**85**), vitexin-2-*O*-α–L rhamnopyranoside (**86**), quercetin-3-*O* xylopyranoside (**81**), quercetin-3-*O*-arabino furanoside (**83**)			[121]
*M. hostilis*	EtOAc	Roots	Indole Alkaloid	*N*,*N*-dimethyltryptamine (**14**)			[122]
*M.artemisiana*	*n*-Hex, MeOH	Leaves and branches	Flavonoids, flavonolignans, glycosylated steroid, triterpene, steroids, indole carboxylate	quercetina-3-*O*-raminosídeo (**87**), miricetina-3-*O*-raminoside (**88**), Euphaline, 3,5,4-trihydroxy-6,7-dimethoxy flavone (**89**), flavonolignana(**90**,**91**), sitosterol-3-*O*-*β*-D glycopyranoside (**21**), lupeol (**22**), steroids sitostenone (**92**), stigmastenone (**93**), campestenone (**94**), campesterol (**23**), stigmasterol (**24**), sitosterol (**25**), methyl indole-3-carboxilate (**95**), indole-3-carboxaldehyde (**96**)			[25]
*M. invisa*	DCM/MeOH	Aerial parts	Fatty aldol ester	17-*O*-triacontanoylheptadecanal (**97**) and *β*-sitosterol (**25**), *α*-amyrine (**98**), lupeol (**22**), 4-*O*-methylepinumisoflavone (**99**), alpinumisoflavone (**100**), betulinic acid (**79**), sitosterol 3-*O*-*β*-D-glucopyranoside (**21**) and epirobinetinidol (**101**)	Antimicrobial activity/*E. coli*, *E. aerogenes*, *S. aureus*, *P. aeruginosa*, *K. pneumonia*, *S. typhi*, *C. albicans*/XTT colorimetric assay	Compound (**97**) and (**101**) were most active against *K. pneumonia* MIC = 64 mg/mL. Overall good activity	[28]
*M. scabrella*	Polysaccharide	Seeds		Sulfated galactomannan (BRS) (**102**)	in vitro/antiviral activity against *Herpes simplex* virus 1 (HSV-1)/plaque reduction method	At concentration 20 μg/mL IC_50_ lesser than 2.5 µg/mL was observed. Marginal activity. Excellent activity.	[123]
					in vitro/Cytotoxic activity/in Vero and MA-104 cells/MTT assay	At the concentrations ≥39 µg/mL BRS reduced by 15% the viability of Vero cells (CC_50_ = 454 µg/mL) At the concentrations 625 µg/mL BRS reduced by 24% the viability of MA-104 cells (CC_50_ > 625 µg/mL). Marginal activity	
*M. somniam*	MeOH	Whole plant	Alkaloid	tryptamine (**103**), *N*-methyl tryptamine (**13**)			[124]
*M. pudica*	EtOAc fration	Whole plant	Flavonoid	2-(2′,6′-dimethyl-3′,4′,5′-alkyl or hydroxy alkyl substituted phenyl)-3-oxy-(alkyl or hydoxy alkyl) 5,7-dihydroxy-chromen-4-one (**104**–**107**)	In vitro/Cytotoxic activity/MTT assay/human lung adenocarcinoma cell line (A549) & human erythroleukemic cell line (K562)	(IC_50_) of against A549 = 76.67 µg/mL and K562 = 287.63 µg/mL, while positive control Doxorubicin A549 = 2.76 µg/mL and K562 = 4.72 µg/mL. Marginal activity	[50]
	HyOH extract	Whole plant	Amino acid	L-mimosine (**108**)	Antioxidant effect/DPPH radical scavenging activity	Compound at 250 μg/mL (IC_50_ = 233.06 μM). Good activity	[34,125]
					In vitro/Cytotoxic activity/daudi cell line/MTT assay	Compound **108**, (IC_50_ = 86.61 μM). Excellent activity	
	CHCl_3_ extracts	Whole plant	Triterpenoid	triterpenoid glycoside (**109**)			[126]
	CHCl_3_ extracts	Whole plant	-	jasmonic acid (**110**), abscisic acid (**111**),			[127]
	EtOH	Whole plant	Flavonoids	5,7,3′,4′-tetrahydroxy-6-*C*-[*β*-D-apiose-(1→4)]-*β*-D-glucopyranosyl flavones (**112**), 7,8,3′,4′-tetrahydroxy-6-*C*-[*α*-L-rhamnopyranosyl-(1→2)]-*β*-D-glucopyranosyl flavone (**113**), 5,7,4′-trihydroxyl-8-*C*-*β*-D-glucopyranosyl flavones (**114**), mimosinamine (**115**), mimosinic acid (**116**), Tyrosin (**117**)			[128]
	EtOH	Whole plant	Flavonoids	5,7,3′,4′-tetrahydroxy-6-*C*-[*β*-D-apiose-(1→4)]-*β*-D-glucopyranosyl flavones (**112**), 6,7,3′,4′-tetrahydroxy-8-*C*-[*α*-L-rhamnopyranosyl-(1→2)]-*β*-D-glucopyranosyl flavone (**118**)			[129]
		Arial parts	Flavonoids	isoquercitrin (**119**), avicularin (**120**), apigenin-7-*O*-D-glucoside (**121**), cassiaoccidentalin B (**122**), orientin (**123**), isoorientin (**124**)			[130]
	MeOH	Leaves		chlorophyllin (**125**)	Antimicrobial activity/*P. aeruginosa*, *E. coli*, *S. aureus*, *K. pneomoniae*, *C. albicans*/well diffusion method	Zone of inhibition at 25 μg/mL conc. *P. aeruginosa* = 12 mm, *E. coli* = 8 mm, *S. aureus* = 14 mm, *K. pneomoniae* = 13 mm, *C. albican* = 9 mm. At 100 μg/mL conc. *P. aeruginosa* = 18 mm, *E. coli =* 13 mm, *S. aureus* = 19 mm, *K. pneomoniae =* 18 mm, *C. albicans* = 13 mm. The streptomycin sulphate and nystatin (standred) at 10 μg/mL showed maximum inhibition 18 mm–19 mm. Good activity	[131]
	EtOAc-benzene (1:9)	Leaves	Phenolic ketone	4-(24′-methoxy-24′-methyl-1′-oxo-5′-n-propyl-tetracosanyl)- phenol (**126**)			[132]
	EtOH	Leaves	Flavonoids	7,3′,4′-triacetoxy-3,8-dimethoxyflavone (**127**), *p*-coumaric acid (**128**), 7,3′,4′-trihydroxy-3,8-dimethoxyflavone (**77**)			[133]
	MeOH	Leaves		mimopudine (**129**)		Responsible for leaves opening	[134]
	MeOH	Leaves		potassium 5-*O*-*β*-D-glucupyranosylgentisate (**130**)		Responsible for leaves closing	[135]
	MeOH	Leaves		mimopudine (**129**), potassium 5-*O*-*β*-D-glucupyranosylgentisate (**130**), potassium L-malate (**131**), magnesium potassium trans-aconitate (**132**), dimethyl ammoniumsalt (**133**)		Responsible for rapid sensitive actions, such as heat and touch. Periodic slow actions, such as nyctinastic actions	[136]
		Fresh leaves		tubulin (**134**)			[137]
	EtOH	Leaves		nor-epinephrine (**135**), d-pinitol (**136**), β-sitosterol (**25**)			[138]
	EtOH	Leaves		5,7,3′,4′-tetrahydroxy-6-*C*-[*β*-D-apiose-(1→4)]-*β*-D-glycopyranosyl flavone (**112**), orientin (**123**), isorientin (**124**), vitexin (**137**), isovitexin (**138**)			[139]
		Roots	Chroman	2-hydroxymethyl-chroman-4-one (**139**)	Antifungal activity/dilution agar plate method/*P. ultimum*, *P. capsici*, *R. solani*, *B. cinerea*, *A. panax* and *S. sclerotiorum*	Compound (**139**) showed good ED_50_ value against *P. capsici* = 35.7 μg/mL, *S. sclerotiorum* = 52.1 μg/mL, *P. ultimum* = 54.9 μg/mL. Overall good activity	[140]
		Roots	Sterolglucoside	stigmasterol (**24**), β-sitosterol (**25**), betulinic acid (**79**), 4-a,24-dimethylcholest-7-en-3*β*-ol-3*β*-D-glucoside (**140**)			[141]
	MeOH	Roots	Diterpenoids	19-*O*-trans-feruloyl-labd-8(17)-en-15,19-diol (**141**), 19-*O*-[(*E*)-3′,4′-dimethoxy cinnamoyl]-labd-8(17)-en- 15,19-diol (**142**)			[142]
		Seeds	Fatty acids	D-xylose (**143**), D-glucuronic acid 4-*O*-(3,5-dihydroxybenzoic acid)-b-D-glucuronide (**144**)			[143]
		Seeds	Cardiac glycosides	bufadienolide (**145**)			[144]
		Stem	Amino acids	mimosine (**108**)			[145]

## Data Availability

The data presented in this study are available on request from the corresponding authors.

## Abbreviations

Butanol = BuOH, Hexane = Hex, Acetone = ACE, Methanol = MeOH, Ethylacetate = EtOAc, Ethanolic = EtOH, Diethyl ether = DEE, Hydroalcohol = HyOH, Aqueous = Aq., Pet. ether = PE, Chloroform = CF, Dicholomethane = DCM, Acetic acid = AA.

## References

[1] Ahuchaogu A.A., Chukwu O.J., Echeme J.O. (2017). Secondary Metabolites from Mimosa Pudica: Isolation, Purification and NMR Characterization. IOSR J. Appl. Chem..

[2] Silva A.S., Araújo S.B., Souza D.C., Silva F.A. (2012). Study of the Cu, Mn, Pb and Zn dynamics in soil, plants and bee pollen from the region of Teresina (PI), Brazil. An. Acad. Bras. Ciências.

[3] Muddiman S., Hodkinson I., Hollis D. (1992). Legume-feeding psyllids of the genus *Heteropsylla* (Homoptera: Psylloidea). Bull. Entomol. Res..

[4] Amalraj T., Ignacimuthu S. (2002). Hyperglycemic effect of leaves of Mimosa pudica Linn. Fitoterapia.

[5] Camargo-Ricalde S.L. (2000). Descripción, distribución, anatomía, composición química y usos de *Mimosa tenuiflora* (Fabaceae-Mimosoideae) en México. Rev. Biol. Trop..

[6] Galinato M.I., Moody K., Piggin C.M. (1999). Upland Rice Weeds of South and Southeast Asia.

[7] Hussain N., Modan M.H., Shabbir S.G., Zaidi S. (1979). Antimicrobial principles in Mimosa hamata. J. Nat. Prod..

[8] Mahmood A., Mahmood A., Qureshi R.A. (2012). Antimicrobial activities of three species of family mimosaceae. Pak. J. Pharm. Sci..

[9] Joyamma V., Rao S., Hrishikeshavan H., Aroor A., Kulkarni D. (1990). Biochemical mechanisms and effects of Mimosa pudica (Linn) on experimental urolithiasis in rats. Indian J. Exp. Biol..

[10] Gupta R., Vairale M., Deshmukh R., Chaudhary P., Wate S.R. (2010). Ethnomedicinal uses of some plants used by Gond tribe of Bhandara district, Maharashtra. Indian J. Tradit. Knowl..

[11] Lemes P.G., Castro A., Zanuncio J. (2014). *Oncideres ocularis* (Coleoptera: Cerambycidae) Girdling *Mimosa bimucronata* (Fabaceae) in Brazil. Fla. Entomol..

[12] Lorenzi H. (1998). Árvores Brasileiras: Manual de Identificação e Cultivo de Plantas Arbóreas Nativas do Brasil.

[13] Jiang Y., Massiot G., Lavaud C., Teulon J.-M., Guéchot C., Haag-Berrurier M., Anton R. (1991). Triterpenoid glycosides from the bark of Mimosa tenuiflora. Phytochemistry.

[14] Ueda M., Takada N., Yamamura S. (2001). Molecular approach to the nyctinastic movement of the plant controlled by a biological clock. Int. J. Mol. Sci..

[15] Fernandes G., Ferrari R.R. (2012). Fruits of *Mimosa foliolosa* (Fabales: Fabaceae) as Sleeping Shelter for *Megachile* (*Pseudocentron*) *botucatuna* (Hymenoptera: Megachilidae). Neotrop. Entomol..

[16] Baggio A.J., Carpanezzi A.A. (1998). Exploração Seletiva do Sub-Bosque: Uma Alternativa para Aumentar a Rentabilidade dos Bracatingais.

[17] Simon M.F., Grether R., de Queiroz L.P., Särkinen T.E., Dutra V.F., Hughes C.E. (2011). The evolutionary history of Mimosa (Leguminosae): Toward a phylogeny of the sensitive plants. Am. J. Bot..

[18] Nworgu F., Egbunike G. (2013). Nutritional Potential of Centrosema pubescens Mimosa invisa and Pueraria phaseoloides Leaf Meals on Growth Performance Responses of Broiler Chickens. Am. J. Exp. Agric..

[19] Erkan G., Şengül K., Kaya S. (2014). Dyeing of white and indigo dyed cotton fabrics with Mimosa tenuiflora extract. J. Saudi Chem. Soc..

[20] Silva T.M.S., dos Santos F.P., Evangelista-Rodrigues A., da Silva E.M.S., da Silva G.S., de Novais J.S., dos Santos F.d.A.R., Camara C.A. (2013). Phenolic compounds, melissopalynological, physicochemical analysis and antioxidant activity of jandaíra (Melipona subnitida) honey. J. Food Compos. Anal..

[21] Vasconcelos do Nascimento C., Albuquerque Pontes Filho R., Artur A., Costa M. (2014). Application of poultry processing industry waste: A strategy for vegetation growth in degraded soil. Waste Manag..

[22] Apolinário V., Dubeux J., Lira M., Luiz R., Mello A., Santos M., Sampaio E.V.S.B., Muir J. (2015). Tree Legumes Provide Marketable Wood and Add Nitrogen in Warm-Climate Silvopasture Systems. Agron. J..

[23] Chiocchio I., Mandrone M., Sanna C., Maxia A., Tacchini M., Poli F. (2018). Screening of a hundred plant extracts as tyrosinase and elastase inhibitors, two enzymatic targets of cosmetic interest. Ind. Crops Prod..

[24] Durgadevi G., Karthika N. (2018). Screening of phytochemicals and pharmacological studies on *Mimosa pudica* L.. Asian J. Innov. Res..

[25] do Nascimento I.A., Braz-Filho R., de Carvalho M.G., Mathias L., de Alcântara Fonseca F. (2012). Flavonoides e Outros Compostos Isolados de *Mimosa artemisiana* Heringer e Paula. Quim. Nova.

[26] Mathew J., Joy J.K., Vazhacharickal P., Sajeshkumar N.K. (2016). Phytochemical Analysis And Invitro Hemostatic Activity Of Mimosa Pudica, Hemigraphis Colorata And Chromolaena Odorata Leaf Extracts. CIBTech J. Pharm. Sci..

[27] Oliveira F.F.M., Barbosa K.M.K.M., de Oliveira G.F., Dantas I.M., Camacho R.G.V. (2007). Micropropagação de Mimosa caesalpiniaefolia Benth. a partir de segmentos nodais e ápices caulinares. Rev. Caatinga.

[28] Nana F., Sandjo L.P., Keumedjio F., Kuete V., Ngadjui B.T. (2012). A new fatty aldol ester from the aerial part of Mimosa invisa (Mimosaceae). Nat. Prod. Res..

[29] Tunna T., Zaidul I., Ahmed Q., Ghafoor K., Al-Juhaimi F., Uddin M., Hasan M., Ferdous S. (2015). Analyses and profiling of extract and fractions of neglected weed Mimosa pudica Linn. traditionally used in Southeast Asia to treat diabetes. S. Afr. J. Bot..

[30] Sutar N., Sutar U.N., Behera B.C. (2009). Antidiabetic activity of the leaves of Mimosa pudica Linn. in albino rats. J. Herb. Med. Toxicol..

[31] Saraswat R., Pokharkar R. (2012). GCMS studies of mimosa pudica. Int. J. PharmTech Res..

[32] Saxena R., Sharma R., Chandra Nandy B., Hardainiyan S. (2017). The Study Of Phenolic Compounds And Antioxidant Potential Of Crude Extract And Fractions Of Mimosa Hamata. Int. Res. J. Pharm..

[33] Almalki M. (2016). In-Vitro Antioxidant Properties of the Leaf Extract of Mimosa pudica Linn. Indian J. Sci. Technol..

[34] Parmar F., Kushawaha N., Highland H., George L.-B. (2015). In vitro antioxidant and anticancer activity of Mimosa pudica linn extract and L-Mimosine on lymphoma daudi cells. Cancer Cell.

[35] Silva M.J.D., Vilegas W., da Silva M.A., de Moura C.F.G., Ribeiro F.A.P., da Silva V.H.P., Ribeiro D.A. (2014). Mimosa (*Mimosa caesalpiniifolia*) prevents oxidative DNA damage induced by cadmium exposure in Wistar rats. Toxicol. Mech. Methods.

[36] Chandarana M., Singh R., Jasrai Y. (2013). Determination of the phenolic and flavonoid contents in Mimosa hamataWilld., as well as their radical scavenging activity. Int. J. Pharmacogn. Phitochem..

[37] Borneo R., León A., Aguirre A., Ribotta P., Cantero J. (2009). Antioxidant capacity of medicinal plants from the Province of Cordoba (Argentina) and their in vitro testing in model food system. Food Chem..

[38] Prathima C., Shashikumara T.T., Jayanthi M.K. (2016). Evaluation of anticonvulsant activity of Mimosa pudica root linn in swiss albino mice. Int. J. Pharm. Pharm. Sci..

[39] Jiang Y., You X.-Y., Fu K.-L., Yin W.-L. (2012). Effects of Extract from Mangifera indica Leaf on Monosodium Urate Crystal-Induced Gouty Arthritis in Rats. Evid. Based Complement. Altern. Med..

[40] Sampaio Octaviano de Souza R., Albuquerque U., Monteiro J., Lúcia Cavalcanti de Amorim E. (2008). Jurema-Preta (*Mimosa tenuiflora* [Willd.] Poir.): A Review of its Traditional Use, Phytochemistry and Pharmacology. Braz. Arch. Biol. Technol..

[41] Patel N.K., Bairwa K., Gangwal R., Jaiswal G., Jachak S.M., Sangamwar A.T., Bhutani K.K. (2015). 2′-Hydroxy flavanone derivatives as an inhibitors of pro-inflammatory mediators: Experimental and molecular docking studies. Bioorgan. Med. Chem. Lett..

[42] Borsato D.M., Prudente A.S., Döll-Boscardin P.M., Borsato A.V., Luz C.F., Maia B.H., Cabrini D.A., Otuki M.F., Miguel M.D., Farago P.V. (2014). Topical anti-inflammatory activity of a monofloral honey of Mimosa scabrella provided by Melipona marginata during winter in Southern Brazil. J. Med. Food.

[43] Bitencourt M., Conceição Jerônimo de Souza Lima M., Torres-Rêgo M., Fernandes J., Silva-Júnior A., Tambourgi D., Maria Zucolotto S., Fernandes-Pedrosa M. (2014). Neutralizing Effects of Mimosa tenuiflora Extracts against Inflammation Caused by Tityus serrulatus Scorpion Venom. BioMed Res. Int..

[44] Cruz M.P., Andrade C.M.F., Silva K.O., de Souza E.P., Yatsuda R., Marques L.M., David J.P., David J.M., Napimoga M.H., Clemente-Napimoga J.T. (2016). Antinoceptive and Anti-inflammatory Activities of the Ethanolic Extract, Fractions and Flavones Isolated from *Mimosa tenuiflora* (Willd.) Poir (Leguminosae). PLoS ONE.

[45] Kumar Vikram P., Malvi R., Jain D. (2015). evaluation of analgesic and anti-inflammatory potential of mimosa pudica linn. Int. J. Curr. Pharm. Res..

[46] Ganguly M., Devi N., Mahanta R., Borthakur M.K. (2008). Effect of *Mimosa pudica* root extract on vaginal estrous and serum hormones for screening of antifetility activity in albino mice. Contraception.

[47] Le Tran Q., Tezuka Y., Ueda J.-Y., Nhan N., Maruyama Y., Begum K., Kim H., Wataya Y., Kim Tran Q., Kadota S. (2003). In Vitro Antiplasmodial Activity of Antimalarial Medicinal Plants Used in Vietnamese Traditional Medicine. J. Ethnopharmacol..

[48] Marimuthu S., Rahuman A., Rajakumar G., Thirunavukkarasu S., Kirthi V., Chidambaram J., Bagavan A., Abduz Zahir A., Elango G., Kamaraj D.C. (2010). Evaluation of green synthesized silver nanoparticles against parasites. Parasitol. Res..

[49] Paul J., Khan S., Mohammed S., Asdaq S. (2010). Wound healing evaluation of chloroform and methanolic extracts of mimosa pudica roots in rats. Int. J. Biol. Med. Res..

[50] Jose J., Dhanya A., Haridas K.R., Kumar T.S., Jayaraman S., Variyar E.J., Sudhakaran S. (2016). Structural characterization of a novel derivative of myricetin from Mimosa pudica as an anti-proliferative agent for the treatment of cancer. Biomed. Pharmacother..

[51] Monção B.N., Araújo Q.B., Silva D.J., Lima J.D., Ferreira M.P., Airoldi P.F., Pessoa C., Citó M.A. (2015). Assessing Chemical Constituents of Mimosa caesalpiniifolia Stem Bark: Possible Bioactive Components Accountable for the Cytotoxic Effect of M. caesalpiniifolia on Human Tumour Cell Lines. Molecules.

[52] Molina M., Contreras C., Tellez-Alcantara P. (1999). Mimosa pudica may possess antidepressant actions in the rat. Phytomedicine.

[53] Saifuddin Khalid M., Shah J., Suresh D.K., Singh R., Narasimha Reddy I.V., Kumar S. (2011). Evaluation of anti-diarrhoeal potential of ethanolic extract of Mimosa pudica leaves. Int. J. Green Pharm..

[54] Balakrishnan N., Bhaskar V., Jayakar B., Sangameswaran B. (2006). Antibacterial activity of *Mimosa pudica*, *Aegle marmelos* and *Sida cordifolia*. Pharmacogn. Mag..

[55] Sowmya A., Ananthi T. (2011). Hypolipidemic activity of Mimosa pudica Linn on butter induced hyperlipidemia in rats. Asian J. Res. Pharm. Sci..

[56] Purkayastha A., Chakravarty P., Dewan B. (2016). hypolipidemic effect of ethanolic extract of mimosa pudica leaves on dyslipidemia following hepatic injury induced by carbon tetrachloride in albino rats. Int. J. Pharm. Sci. Res..

[57] Kumaresan R., Veerakumar S., Elango V. (2015). A study on hepatoprotective activity of Mimosa pudica in albino rats. Int. J. Pharm. Phytochem. Res..

[58] Lin L.-C., Chiou C.-T., Cheng J.-J. (2011). 5-Deoxyflavones with cytotoxic activity from Mimosa diplotricha. J. Nat. Prod..

[59] Jain S., Jain R., Vlietinck A. (2004). In vivo and in vitro antimicrobial efficacy of Mimosa hamata. Indian J. Biotechnol..

[60] Mehta B.K., Savita Sharma K., Dubey A. (1988). 4-Ethylgallic acid from two Mimosa species. Phytochemistry.

[61] Mehmood N., Zubair M., Rızwan K., Rasool N., Shahid M., Ahmad V. (2012). Antioxidant, antimicrobial and phytochemical analysis of *Cichorium intybus* seeds extract and various organic fractions. Iran. J. Pharm. Res..

[62] Aslam F., Riaz M., Zubair M., Rizwan K., Abbas M., Bukhari T.H., Bukhari I.H. (2012). Antioxidant, haemolytic activities and GC-MS profiling of Carissa carandas roots. Int. J. Phytomed..

[63] Bari M.N., Zubair M., Rizwan K., Rasool N., Bukhari I.H., Akram S., Bokhari T.H., Shahid M., Hameed M., Ahmad V.U. (2012). Biological activities of Opuntia Monacantha cladodes. J. Chem. Soc. Pak..

[64] Imran M., Rasool N., Rizwan K., Zubair M., Riaz M., Zia-Ul-Haq M., Rana U.A., Nafady A., Jaafar H.Z. (2014). Chemical composition and Biological studies of Ficus benjamina. Chem. Cent. J..

[65] Rizwan K., Zubair M., Rasool N., Mahmood A., Ercisli S., Tareen R.B., Zia-Ul-Haq M. (2016). Compositional studies and antioxidant potential of Fruit of *Zizyphus oxyphylla* EDGEW. Oxid. Commun..

[66] Ashraf S.N., Zubair M., Rizwan K., Tareen R.B., Rasool N., Zia-Ul-Haq M., Ercisli S. (2014). Compositional studies and Biological activities of *Perovskia abrotanoides* Kar. oils. Biol. Res..

[67] Rasool N., Tehseen H., Riaz M., Rizwan K., Zubair M., Mahmood Y., Iqbal M., Bukhari I.H. (2013). Cytotoxicity studies and antioxidant potential of Acacia nilotica roots. Int. J. Chem. Biochem. Sci..

[68] Rasool N., Afzal S., Riaz M., Rashid U., Rizwan K., Zubair M., Ali S., Shahid M. (2013). Evaluation of antioxidant activity, cytotoxic studies and GC-MS profiling of matthiola incana (stock flower). Legume Res. Int. J..

[69] Khan S.A., Rasool N., Riaz M., Nadeem R., Rashid U., Rizwan K., Zubair M., Bukhari I.H., Gulzar T. (2013). Evaluation of Antioxidant and Cytotoxicity Studies of Clerodendrum inerme. Asian J. Chem..

[70] Zubair M., Rizwan K., Rashid U., Saeed R., Saeed A.A., Rasool N., Riaz M. (2017). GC/MS profiling, in vitro antioxidant, antimicrobial and haemolytic activities of *Smilax macrophylla* leaves. Arab. J. Chem..

[71] Zia-Ul-Haq M., Stanković M.S., Rizwan K., Feo V.D. (2013). Grewia asiatica L., a food plant with multiple uses. Molecules.

[72] Zubair M., Bibi Z., Rizwan K., Rasool N., Zahoor A.F., Riaz M. (2013). In Vitro *Antimicrobial* and Haemolytic Studies of *Bambusa arundinaceae* leaves. J. Appl. Pharm. Sci..

[73] Riaz M., Rasool N., Bukhari I.H., Shahid M., Zubair M., Rizwan K., Rashid U. (2012). In vitro antimicrobial, antioxidant, cytotoxicity and GC-MS analysis of Mazus goodenifolius. Molecules.

[74] Rizwan K., Zubair M., Rasool N., Riaz M., Zia-Ul-Haq M., de Feo V. (2012). Phytochemical and biological studies of Agave attenuata. Int. J. Mol. Sci..

[75] Riaz M., Rasool N., Bukhari I., Rizwan K., Zubair M., Javed F., Altaf A., Qayyum H. (2012). Antioxidant, Antimicrobial Activity and GC-MS analysis of *Russelia equsetiformis* Essential Oils. Oxid. Commun..

[76] Sharma P.V., Ahmad Z.A. (1987). Present status of research on genus: Mimosa. Anc. Sci. Life.

[77] Singh R., Jasrai Y.T. (2012). *Mimosa hamata* (Willd.), a plant with antipathogenic properties. Int. J. Med. Aromat. Plants.

[78] Jasuja N.D., Saxena R., Chandra S., Bhargava S., Joshi S. (2014). Pharmacological evaluation of an ethnomedicinal and endangered desert plant: *Mimosa hamata*. J. Biol. Sci..

[79] Muhammad G., Hussain M.A., Jantan I., Bukhari S.N.A. (2016). *Mimosa pudica* L., a high-value medicinal plant as a source of bioactives for pharmaceuticals. Compr. Rev. Food Sci. Food Saf..

[80] Sanaye M., Joglekar C., Pagare N. (2015). *Mimosa*—A brief overview. J. Pharmacogn. Phytochem..

[81] Majeed I., Rizwan K., Ashar A., Rasheed T., Amarowicz R., Kausar H., Zia-Ul-Haq M., Marceanu L.G. (2021). A Comprehensive Review of the Ethnotraditional Uses and Biological and Pharmacological Potential of the Genus Mimosa. Int. J. Mol. Sci..

[82] Haddad H., Seyed Hoseini E., Nikzad H., Hossein Aarabi M. (2012). Pharmacological properties of medicinal herbs by focus on secondary metabolites. Life Sci. J..

[83] Oliveira L.M.B., Macedo I.T.F., Vieira L.S., Camurça-Vasconcelos A.L.F., Tomé A.R., Sampaio R.A., Louvandini H., Bevilaqua C.M.L. (2013). Effects of Mimosa tenuiflora on larval establishment of Haemonchus contortus in sheep. Vet. Parasitol..

[84] Racadio S.P. (2016). The Medicinal Prospects of Makahiya (Mimosa Pudica Linn) Plant. Adv. Life Sci..

[85] Ahuchaogu A., Chukwu J., Obike A., Ugonna Oha T., Bull J., Echeme O. (2017). Quantitative Determination of Secondary Metabolites and Antibacterial Activity of Mimosa Pudica. Int. J. Med. Plants Nat. Prod..

[86] Sheeba D.G., Gomathi K.S., Citarasu D. (2015). Anti-Mycobacterial and Phytochemical Investigation of Methanol Extracts of Few Medicinal Plants. J. Chem. Pharm. Sci..

[87] Mahadevan M.V., Ramaswamy R.S., Banumathi V. (2016). Mimosa pudica exerts neuroprotection against mpp+ induced neurotoxicity in shsy5y cell lines-an in vitro model of anti-parkinsonism. Int. J. Pharm. Pharm. Sci..

[88] Ramesh S., Karthikeyan K., Chandran C. (2017). Photochemical screening and pharmacognostic studies on *Mimosa pudica* L. (*Sensitive plant*). Int. J. Fauna Biol. Stud..

[89] Chinnathambi A., Sathasivam A. Analysis of the phytochemical constituents of Mimosa pudica and determination of their antimicrobial activity. Proceedings of the Biotech Congress.

[90] Harborne J.B., Williams C.A. (2001). Anthocyanins and other flavonoids. Nat. Prod. Rep..

[91] Nagarajan K., Saravanararaja M., Aruna Devi P.S. (2015). Antibacterial Efficiency of Fabaceae Plants of a Tropical Freshwater Lake. ScieXplore Int. J. Res. Sci..

[92] Lee Y.H., Choo C., Watawana M.I., Jayawardena N., Waisundara V.Y. (2015). An appraisal of eighteen commonly consumed edible plants as functional food based on their antioxidant and starch hydrolase inhibitory activities. J. Sci. Food Agric..

[93] Ittiyavirah S.P., Pullochal I. (2014). Adaptogenic and nootropic activity of Mimosa pudica in albino wistar rats. Int. J. Nutr. Pharmacol. Neurol. Dis..

[94] Ao S., Kc O., Ukwueze S. (2015). Evaluation of the antidiabetic potential of ethanol root extract of Mimosa pigra Linn (Fabaceae) in alloxan-induced diabetic albino rats. Int. J. Curr. Res..

[95] Shorinwa Olusayo A., Nnamdi E., Afieroho Ozadheoghene E. (2016). Evaluation of mimosa pigra roots on haematological and biochemical parameters of albino rats. World J. Pharm. Res..

[96] Rosado-Vallado M., Brito-Loeza W., Mena-Rejon G., Quintero-Marmol E., Flores-Guido J. (2000). Antimicrobial activity of *Fabaceae* species used in Yucatan traditional medicine. Fitoterapia.

[97] Saxena R., Sharma R., Nandy B., Jasuja D.N. (2014). Qualitative and quantitative estimation of bioactive compounds in *Mimosa hamata*. Int. J. Pharm. Pharm. Sci..

[98] Manosroi J., Zaruwa M., Manosroi A. (2011). Potent Hypoglycemic Effect of Nigerian Anti-Diabetic Medicinal Plants. J. Complement. Integr. Med..

[99] Jiménez N., Carrillo-Hormaza L., Pujol A., Álzate F., Osorio E., Lara-Guzman O. (2015). Antioxidant capacity and phenolic content of commonly used anti-inflammatory medicinal plants in Colombia. Ind. Crops Prod..

[100] Seraglio S.K.T., Valese A.C., Daguer H., Bergamo G., Azevedo M.S., Gonzaga L.V., Fett R., Costa A.C.O. (2016). Development and validation of a LC-ESI-MS/MS method for the determination of phenolic compounds in honeydew honeys with the diluted-and-shoot approach. Food Res. Int..

[101] Nandipati M.C., Sumalatha G., Baburao C., Babu J.R., Sridevi C. (2014). Antitumor activity of mimosa rubicaulis lam against ehrlich ascites carcinoma in swiss albino mice. Int. J. Pharm. Sci. Res..

[102] Wadood A., Ghufran M., Jamal S.B., Naeem M., Khan A., Ghaffar R. (2013). Phytochemical analysis of medicinal plants occurring in local area of Mardan. Biochem. Anal Biochem..

[103] Dominguez X.A., Garcia G.S., Williams H.J., Ortiz C., Scott A.I., Reibenspies J.H. (1989). Kukulkanins A and B, new chalcones from *Mimosa tenuefolia*. J. Nat. Prod..

[104] Bautista E., Calzada F., Ortega A., Yépez-Mulia L. (2011). Antiprotozoal activity of flavonoids isolated from *Mimosa tenuiflora* (Fabaceae-Mimosoideae). J. Mex. Chem. Soc..

[105] León L., Maldonado E., Cruz A., Ortega A. (2004). Tenuiflorins A-C: New 2-Phenoxychromones from the Leaves of *Mimosa tenuiflora*. Planta Med..

[106] Gardner D., Riet-Correa F., Lemos D., Welch K., Pfister J., Panter K. (2014). Teratogenic effects of Mimosa tenuiflora in a rat model and possible role of N-methyl-and N, N-dimethyltryptamine. J. Agric. Food Chem..

[107] Meckes-Lozoya M., Lozoya X., Marles R.J., Soucy-Breau C., Sen A., Arnason J.T. (1990). N, N-dimethyltryptamine alkaloid in *Mimosa tenuiflora* bark (tepescohuite). Arch. Investig. Med..

[108] Anton R., Jiang Y., Weniger B., Beck J., Rivier L. (1993). Pharmacognosy of *Mimosa tenuiflora* (willd.) poiret. J. Ethnopharmacol..

[109] Jiang Y., Weniger B., Haag-Berrurier M., Anton R., Beck J.P., Italiano L. (1992). Effects of saponins from *Mimosa tenuiflora* on lymphoma cells and lymphocytes. Phytother. Res..

[110] Vepsäläinen J.J., Auriola S., Tukiainen M., Ropponen N., Callaway J. (2005). Isolation and characterization of yuremamine, a new phytoindole. Planta Med..

[111] Calvert M.B., Sperry J. (2015). Bioinspired total synthesis and structural revision of yuremamine, an alkaloid from the entheogenic plant *Mimosa tenuiflora*. Chem. Commun..

[112] Okonkwo C.J., Njoku O.U., Okonkwo T.J., Afieroho O.E., Proksch P. (2016). Two new acylated flavonol glycosides from *Mimosa pigra* L. leaves sub-family *Mimosoideae*. Future J. Pharm. Sci..

[113] Englert J., Weniger B., Lobstein A., Anton R., Krempp E., Guillaume D., Leroy Y. (1995). Triterpenoid saponins from *Mimosa pigra*. J. Nat. Prod..

[114] Rakotomalala G., Agard C., Tonnerre P., Tesse A., Derbré S., Michalet S., Hamzaoui J., Rio M., Cario-Toumaniantz C., Richomme P. (2013). Extract from *Mimosa pigra* attenuates chronic experimental pulmonary hypertension. J. Ethnopharmacol..

[115] Santos M., Moura L., Mendes M., Arcanjo D., Monção N., Araújo B., Lopes J., Silva-Filho J., Fernandes R., Oliveira R. (2015). Hypotensive and vasorelaxant effects induced by the ethanolic extract of the *Mimosa caesalpiniifolia Benth*.(*Mimosaceae*) inflorescences in normotensive rats. J. Ethnopharmacol..

[116] Silva M., Carvalho A., Rocha C., Vilegas W., Silva M., Gouvêa C. (2014). Ethanolic extract of *Mimosa caesalpiniifolia* leaves: Chemical characterization and cytotoxic effect on human breast cancer MCF-7 cell line. S. Afr. J. Bot..

[117] Jain R., Arora R., Jain S. (1997). Saponins from the Roots of *Mimosa hamata* Willd. ChemInform.

[118] Jain R., Arora R., Alam S., Jain S. (1997). Pharmacological evaluation of *Mimosa hamata* roots. Fitoterapia.

[119] Jain R., Jain S. (2015). Studies on Antimicrobial and Antioxidant Potentials of Triterpenoidal Saponins from Mimosa Hamata Willd. Int. J. Pharm. Phytopharm. Res..

[120] Chiou C.-T., Shen C.-C., Tsai T.-H., Chen Y.-J., Lin L.-C. (2016). Meroterpenoids and Chalcone-Lignoids from the Roots of *Mimosa diplotricha*. J. Nat. Prod..

[121] Camargo L.M.D.M., Férézou J.-P., Tinoco L.W., Kaiser C.R., Costa S.S. (2012). Flavonoids from *Mimosa xanthocentra* (Leguminosae: Mimosoideae) and molecular modeling studies for isovitexin-2″-O-α-l-rhamnopyranoside rotamers. Phytochem. Lett..

[122] Pachter I.J., Zacharias D.E., Ribeiro O. (1959). Indole alkaloids of *Acer saccharinum* (the Silver Maple), *Dictyoloma incanescens*, *Piptadenia colubrina*, and *Mimosa hostilis*. J. Org. Chem..

[123] Chrestani F., Sierakowski M.R., de Andrade Uchoa D.E., Nozawa C., Sassaki G.L., Gorin P.A.J., Ono L. (2009). In vitro antiherpetic and antirotaviral activities of a sulfate prepared from *Mimosa scabrella* galactomannan. Int. J. Biol. Macromol..

[124] Gupta M., Arias T., Etheart J., Hatfield G. (1979). The occurrence of tryptamine and N-methyltryptamine in *Mimosa somnians*. J. Nat. Prod..

[125] Thompson J.F., Morris C.J., Smith I.K. (1969). New naturally occurring amino acids. Annu. Rev. Biochem..

[126] Chukwu O.J., Ahuchaogu A.A., Ukaogo P., Obike A., Echeme J.B.O. (2017). Antifungal Activity of *Mimosa pudica*, Isolation and NMR Characterization of Bioactive Components. Asian J. Chem. Sci..

[127] Tsurumi S., Asahi Y. (1985). Identification of jasmonic acid in *Mimosa pudica* and its inhibitory effect on auxin-and light-induced opening of the pulvinules. Physiol. Plant..

[128] Yuan K., Lü J., Yin M. (2006). Chemical constituents of C-glycosylflavones from *Mimosa pudica*. Yao Xue Xue Bao Acta Pharm. Sin..

[129] Yuan K., Lu J.L., Jia A., Zhu J.X. (2007). Two new C-glycosylflavones from *Mimosa pudica*. Chin. Chem. Lett..

[130] Misra A., Tiwari H. (1971). Constituents of roots of *Boerhaavia diffusa*. Phytochemistry.

[131] Rajalakshmi K., Banu N. (2016). Antimicrobial activity of natural chlorophyllin from endangered medicinal plant *Mimosa pudica* L.. Int. J. Pharm. Pharm. Sci..

[132] Josewin B., Ramachandrapai M., Suseelan M. (1999). A new phenolic ketone from the leaves of *Mimosa pudica* Linn. Indian J. Chem..

[133] Kirk L.F., Møller M.V., Christensen J., Stærk D., Ekpe P., Jaroszewski J.W. (2003). A 5-deoxyflavonol derivative in *Mimosa pudica*. Biochem. Syst. Ecol..

[134] Ueda M., Yamamura S. (1999). Leaf-opening substance of *Mimosa pudica* L.; chemical studies on the other leaf movement of mimosa. Tetrahedron Lett..

[135] Ueda M., Yamamura S. (1999). Leaf-closing substance of *Mimosa pudica* L.; chemical studies on another leaf-movement of mimosa II. Tetrahedron Lett..

[136] Ueda M., Yamamura S. (1999). The chemistry of leaf-movement in *Mimosa pudica* L.. Tetrahedron.

[137] Pal M., Roychaudhury A., Pal A., Biswas S. (1990). A novel tubulin from *Mimosa pudica*: Purification and characterization. Eur. J. Biochem..

[138] Khare C. (2004). Encyclopedia of Indian Medicinal Plants: Rational Western Therapy, Ayurvedic and Other Traditional Usage, Botany.

[139] Zhang J., Yuan K., Zhou W.-l., Zhou J., Yang P. (2011). Studies on the active components and antioxidant activities of the extracts of *Mimosa pudica* Linn. from southern China. Pharmacogn. Mag..

[140] Kang J.G., Shin S.Y., Kim M.J., Bajpai V., Maheshwari D.K., Kang S.C. (2004). Isolation and Anti-fungal Activities of 2-Hydroxymethyl-chroman-4-one Produced by *Burkholderia* sp. MSSP. J. Antibiot..

[141] Dinda B., Ghosh B., Arima S., Sato N., Harigaya Y. (2006). Steroids and terpenoid from *Mimosa pudica* roots. J. Indian Chem. Soc..

[142] Shu W.-J., Ho J.-C. (2013). Two new antimicrobial diterpenoids from the roots of *Mimosa pudica*. J. Chin. Med..

[143] Chatterjee A., Pakrashi S.C. (1991). The Treatise on Indian Medicinal Plants.

[144] Yadava R., Yadav S. (2001). A novel bufadienolide from the seeds of *Mimosa pudica* Linn. Asian J. Chem..

[145] Zaware B., Chaudhari S., Shinde M. (2014). An overview of *Mimosa pudica* Linn. Chem. Pharmacol. Profile. Res. J. Pharm. Biol. Chem. Sci..

[146] Yeung A.W.K., Tzvetkov N.T., Georgieva M.G., Ognyanov I.V., Kordos K., Jóźwik A., Kühl T., Perry G., Petralia M.C., Mazzon E. (2020). Reactive Oxygen Species and Their Impact in Neurodegenerative Diseases: Literature Landscape Analysis. Antioxid. Redox Signal..

[147] Sueishi Y., Nii R. (2020). A comparative study of the antioxidant profiles of olive fruit and leaf extracts against five reactive oxygen species as measured with a multiple free-radical scavenging method. J. Food Sci..

[148] Gupta R.K., Rathi R.B. (2021). Evaluation of Anticancer Action of Martynia annua Linn Root Extract on Different Human Cancer Cell Lines. J. Pharm. Res. Int..

[149] Khan T., Ali M., Khan A., Nisar P., Jan S.A., Afridi S., Shinwari Z.K. (2020). Anticancer plants: A review of the active phytochemicals, applications in animal models, and regulatory aspects. Biomolecules.

